# Investigation of printed slot antennas based on Euclidean geometries

**DOI:** 10.1038/s41598-021-83772-2

**Published:** 2021-02-24

**Authors:** Yaqeen S. Mezaal

**Affiliations:** Medical Instrumentation Engineering Department, Al-Esraa University College, Baghdad, Iraq

**Keywords:** Engineering, Electrical and electronic engineering

## Abstract

Euclidean and fractal terms are mathematically and physically important terms in antenna design, but rarely reported studies had discussed these terms together in antenna design in their texts. This paper first gives an overview of Euclidean and fractal antennas with useful and satisfactory facts. Four printed slot antennas are then studied using Euclidean slot shapes printed in the ground plane with and without Euclidean patches using FR4 substrate. These antennas are employed to investigate their suitability as simple alternatives to complicated fractal geometries and their specific formulas. Parametric analyses with feedline lengths and patch scaling aspects are adopted to generate single, dual, and multiband responses. These parametric studies provide different outcomes and choices for antenna electrical specifications suitable for various wireless applications. It is clear that inserting Euclidean patches to the printed slot in the ground plane influence inducing multiple operating bands as similar as multiband fractal antenna, but without using specific formulas or complicated outlines. All proposed antennas have low-profile topologies, good compactness, and more competitive electrical specifications than many reported fractal antennas. The simulations of the proposed printed slot antennas are in good compatibility with the measurements.

## Introduction

The continuous requirements for the antennas that have the properties of small volume, low weight and simplicity of manufacturing using printed-circuit technology, led to many designed antenna outlines for different wireless applications. Due to the increasing necessities of personal and mobile communications, the requirement for more compact antennas with sensible performances has brought the microstrip technology to the forefront^1, 2^. Recently, multifunctional wireless communication systems are continually requested. Typically, each antenna functions at a single frequency band, where diverse antennas are used for a particular band application. This may cause a problem due to the limited space; especially, most manufacturers tend to produce compact devices and systems. A dual/multiband antenna can be employed to overcome this, where a distinct antenna can operate at numerous frequency bands^3, 4^. Slotted patch structures with diverse outlines were used to design dualband/multiband antennas. In the earlier decades, these antennas were illustrious for their desirable properties like broader bandwidth, less interaction via surface waves, superior isolation, and trivial radiation from feed networks. Additionally, they have the characteristics of compactness to be integrated within many planar circuits and wireless schemes. Therefore, slotted patch antennas have been studied by countless scholars all over the world^4^.

Traditional antenna designs are based on Euclidean geometries, but innovative and non-Euclidean designs have been developed as in fractal geometries. Fractal shaped antennas have the possibilities to give several remarkable characteristics that relate to their geometrical features. Fractal formations are typically composed of manifold copies of themselves at diverse scales, and the initiator and iteration number can define the size of a fractal. The applied fractal for antenna design can be typically a pre-fractal or a quasi-fractal but not a precisely fractal geometry with unlimited scale. Accordingly, designers can employ a quasi or semi fractal outline with limited iterations for a particular multiband antenna. All frequency bands are in relation to an actual scale of the fractal^5^. The structures of fractal antennas can be random or deterministic. A random fractal is systematized randomly based on infinite iterative orders, and its shapes are from nature as in tree fractal antennas^6^. Deterministic fractal can be created after regular iterative categories of fractal antennas, as in Koch, Sierpinski, Hilbert, Minkowski, and Cantor antennas. Fractal antennas have a particularity of enhanced electrical to physical size ratio as compared with customary antennas^7, 8^. However, not all fractal antennas are better than Euclidean antennas in terms of electrical specifications. For instance, the genetic algorithm has been adopted to optimize the bandwidth and efficiency of wire antennas and reducing their resonance frequency as reported in^9^, including second-order Koch-like pre-fractal antennas and Euclidean antennas based on zig-zag and meander curves. The performance of Euclidean antennas was at all times superior to Koch-like structures.

In^10^, a miniature dualband slotted patch antenna was simulated using a high-frequency full-wave electromagnetic simulator based on finite element routine. The projected antenna was manufactured employing LPKF machine on fiberglass epoxy polymer resin material substrate, while the prototyped antenna performance was determined in a typical far-field anechoic measurement chamber. The measurements of impedance bandwidths of 12.26%, 8.24%, and 3.08% for frequency ranges of (14.3–16.2 GHz), (17.4–18.9 GHz) and (19.2–19.8 GHz) respectively, were realized by this designed antenna prototype. In^11^, a new dualband printed antenna using a slotted patch and a defected ground structure (DGS) was presented. By introducing an I-shaped slot into the patch and defecting the ground plane, triple-mode resonance for accomplishing dualband operation with a particularly wide bandwidth for the higher band was realized. The bandwidth measurements are 100 MHz (2.38–2.49 GHz) and 2.94 GHz (3.4–6.34 GHz) for the minor and higher bands, correspondingly, that correspond satisfactorily with the simulated results. Seljuk star-shaped slotted microstrip patch antenna was printed on a rectangular shape glass epoxy FR4 substrate. Probe feed was employed in this antenna for multiband performance. The multiband performance was investigated using dual-slot iterations. The projected antenna covers multiband within 1.6–5.9 GHz frequency range^12^.A compact antenna consisting of an elliptical patch, a slotting line, and a coaxial feedline is proposed in^13^. This design comprises a square patch and the capability to cover multiple bands in a 1–15 GHz range. The simulation results of square patch with slotted line arrangement with different slot size and with the modification in slot geometry of these different arrangements are presented. The slotted square patch antenna is designed with an RT-Duroid substrate (ε_r_ = 2.22, h = 0.37 mm). Simulation results of input reflection and polar radiation pattern are investigated. In^14^, a printed wide-slot antenna with wideband frequency response has been experimentally investigated based on a modified L-shaped microstrip line for exciting the square slot. It has a horizontal line, a square patch, and a vertical line. This antenna's overall surface area is 80 × 80 mm^2^ with a wide bandwidth of 3510 MHz and an applicable gain of 4.6 dBi that is suitable for many wireless systems. A rectangular CPW folded slot antenna with a dualband response has been considered for the Global Navigation Satellite Systems (GNSS) applications, as stated in^15^. This antenna can be operated for each band of the GNSS Systems (L1-L2-L5-E5-E6-G1-G2) and its supreme gain has been within 0.2–2.9 dB range for the band of 1200–1800 MHz. A circular slot antenna using offset microstrip-fed line has been presented in^16^. This antenna shows dualband response under operating frequency bands of 1.83–2.73 GHz and 5.36–7.63 GHz with impedance bandwidth of 39.5% and 34.9%, respectively. The bands have been valid for UMTS, PCS, ISM, IMT- 2000, RFID, and Bluetooth in addition to WLAN applications. In^17^, a group of small thin-wire antennas based on Euclidean parts produced genetically had been presented. These small thin-wire antennas were electrically in superior performance as compared with some sets of Sierpinski pre-fractal monopoles of the identical electrical size at specified resonance.

In this paper, four printed slot antennas have been introduced based on Euclidean shapes to produce single, dual and multiband frequencies. One of these printed slot antennas is circle based slot structure and the other is octagon based slot structure. These antennas produce dual and single-band responses for each of them, respectively. On the other hand, the other two printed slot antennas using a circle-based slot structure with an octagon patch and an octagon-based slot structure with a circle patch are also presented. Parametric investigations are done to examine the antenna responses as a function of microstrip feed length and patch radius length to acquire the suitable electrical specifications. Accordingly, this study gives an impression that Euclidean antennas using the printed slot approach can be competitors and alternatives to the fractal antennas without using complicated structures, iteration and formulas.

## Antenna design

**Figure 1:**
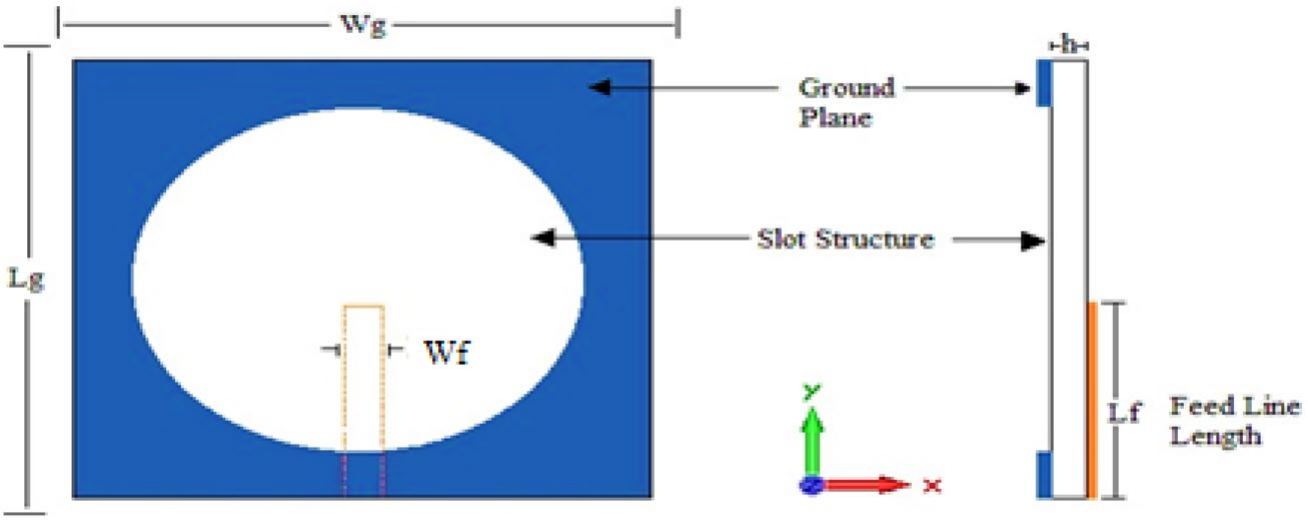
Antenna1 topology.

All the depicted antennas are devices based on Euclidean slots with and without Euclidean patches etched on the ground plane. All these structures have dimensions of a substrate (Wg × Lg) of 50 × 50 ${\mathrm{mm}}^{2}$. Single microstrip feed coupled in the upper plane of the substrate has been employed in the antenna design. The first projected printed slotted antenna is based on the circle shape, as shown in Fig. 1. The slot structure has a radius (R) of 20 mm. The microstrip feeder length (Lf) and width (Wf) are 23 and 3 mm, respectively.

**Figure 2:**
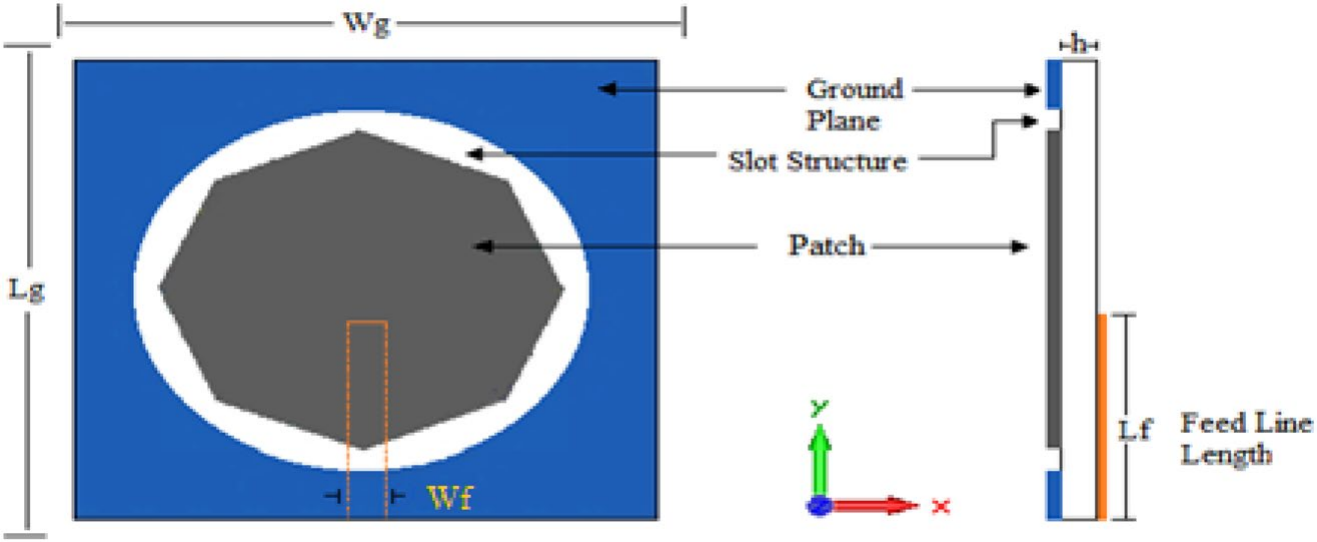
Antenna2 topology.

Figure 2 shows the second printed slot antenna. This antenna is the same antenna depicted in Fig. 1, but a central octagonal patch has been added to the slot structure. The radius of the octagon patch is 18 mm. The values of Lf and Wf are 29 and 3 mm, respectively.

**Figure 3:**
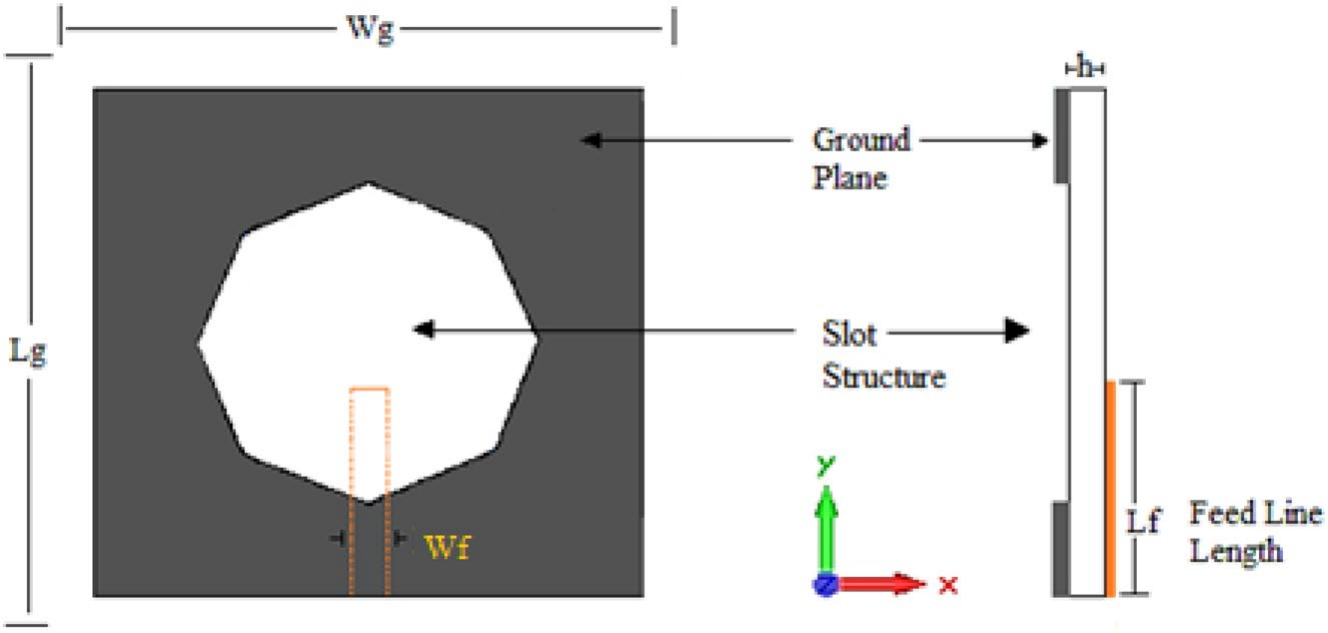
Antenna3 topology.

Figure 3 shows the third printed slot antenna, which is based on an octagonal shape. The radius of the octagonal slot is 15.67 mm. Lf and Wf magnitudes are 19 and 3 mm, respectively.

**Figure 4:**
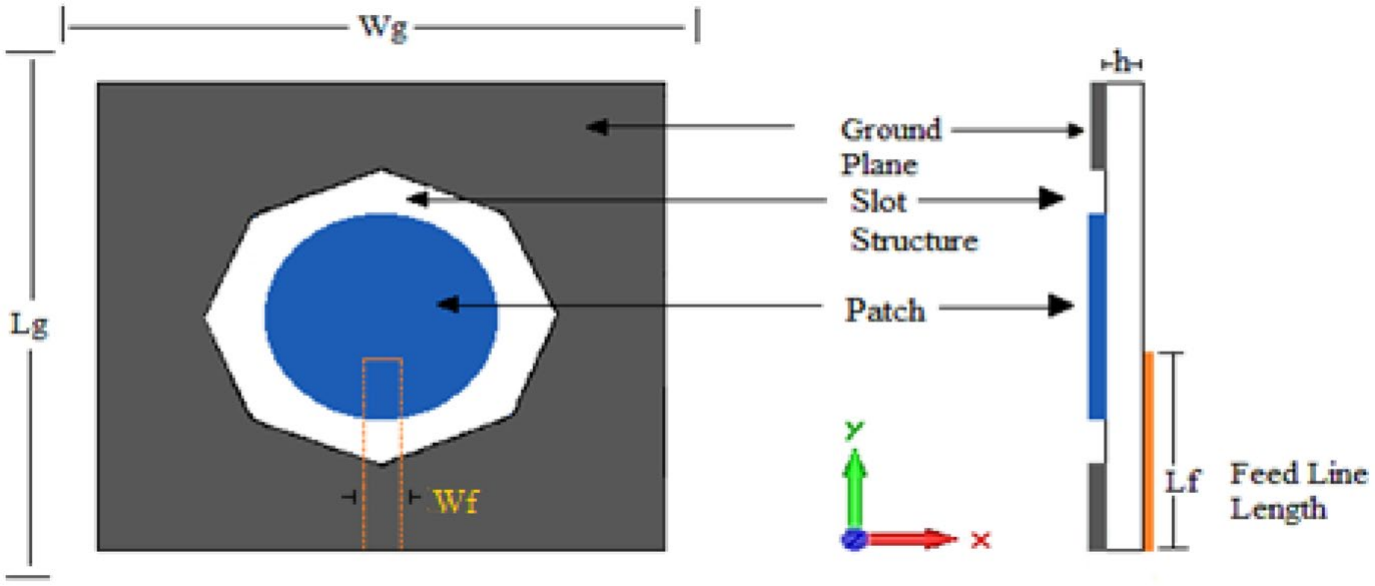
Antenna4 topology.

Figure 4 depicts the fourth printed slot antenna. This antenna is the same antenna shown in Fig. 3, but a circular patch is added to the slot structure. The radius of the circular patch is 10 mm. The values of Lf and Wf are 17 and 3 mm, respectively.

The substrate width and length of above Euclidean antennas are equal (Wg = Lg), and the following equations can be used to calculate the fundamental frequencies (F1, F2, F3, and F4) for Antenna1, Antenna2, Antenna3, and Antenna4:

1 $$\mathrm{F}1=\frac{0.592 c}{\mathrm{Lg} \sqrt{\frac{{\varepsilon }_{r}+1}{2}}}$$

2 $$\mathrm{F}2=\frac{0.559 c}{\mathrm{Lg }\sqrt{\frac{{\varepsilon }_{r}+1}{2}}}$$

3 $$\mathrm{F}3=\frac{1.095 c}{\mathrm{Lg} \sqrt{\frac{{\varepsilon }_{r}+1}{2}}}$$

4 $$\mathrm{F}4=\frac{0.6 c}{\mathrm{Lg} \sqrt{\frac{{\varepsilon }_{r}+1}{2}}}$$

where ${\varepsilon }_{r}$ is the relative dielectric constant, and c is light speed. In terms of guided wavelength (${\lambda }_{g}$), the anticipated antennas have substrate size of about 0.592 ${\lambda }_{g}$ × 0.592 ${\lambda }_{g}$, 0.559 ${\lambda }_{g}$ × 0.559${\lambda }_{g}$, 1.095 ${\lambda }_{g}$ × 1.095 ${\lambda }_{g}$ and 0.6${\lambda }_{g}$ × 0.6 ${\lambda }_{g}$ at their fundamental resonant frequencies of 2.7, 2.55, 5.2 and 2.74 GHz for Antenna1, Antenna2, Antenna3 and Antenna4 respectively. The guided wavelength has been computed based on^18^.

## Results and discussion

Each printed slot antenna was simulated using Computer Simulation Technology (CST) electromagnetic simulator. This simulator has accurate, capable computational solutions for antenna modeling and electromagnetic performance investigation. Also, it has excellent possibilities to produce parametric studies for any antenna dimension term or any resultant electrical parameter in good simulation running time.

**Figure 5:**
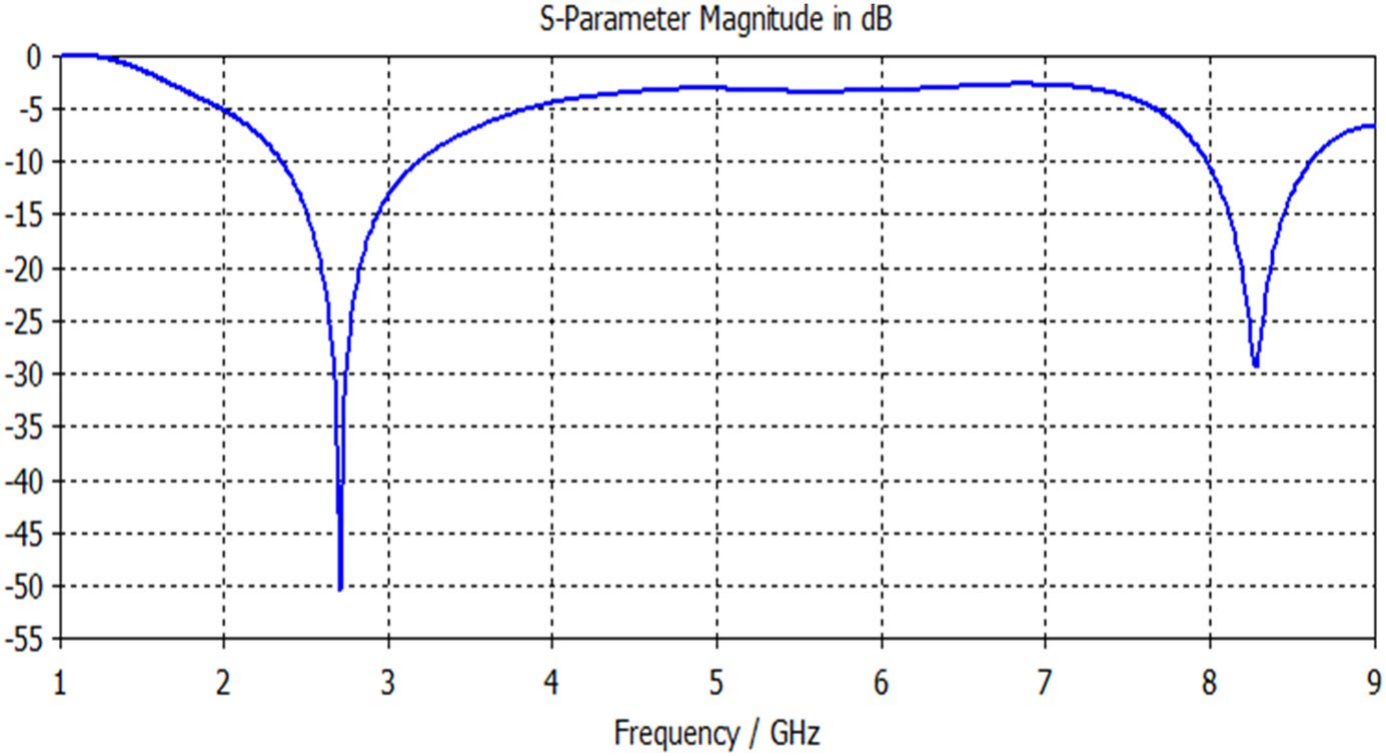
S11 response of Antenna1.

For the first proposed printed circle based slot antenna illustrated in Fig. 1, its input reflection response is determined within the swept frequency range from 1 to 9 GHz using a substrate of 4.4 dielectric constant and 1.6 mm dielectric thickness. As shown in Fig. 5, the circle based slot antenna offers a dual-band response using a feedline length of 23 mm. Table 1 explains the electrical specifications of this antenna. This antenna is suitable for WLAN and satellite systems.

**Table 1:** Electrical properties of Antenna1 response.

F1 (GHz)	F2 (GHz)	S11 (F1) dB	S11 (F2) dB	BW1 (GHz)	BW2 (GHz)
2.7	8.3	− 50	− 29.2	0.41	0.34

**Figure 6:**
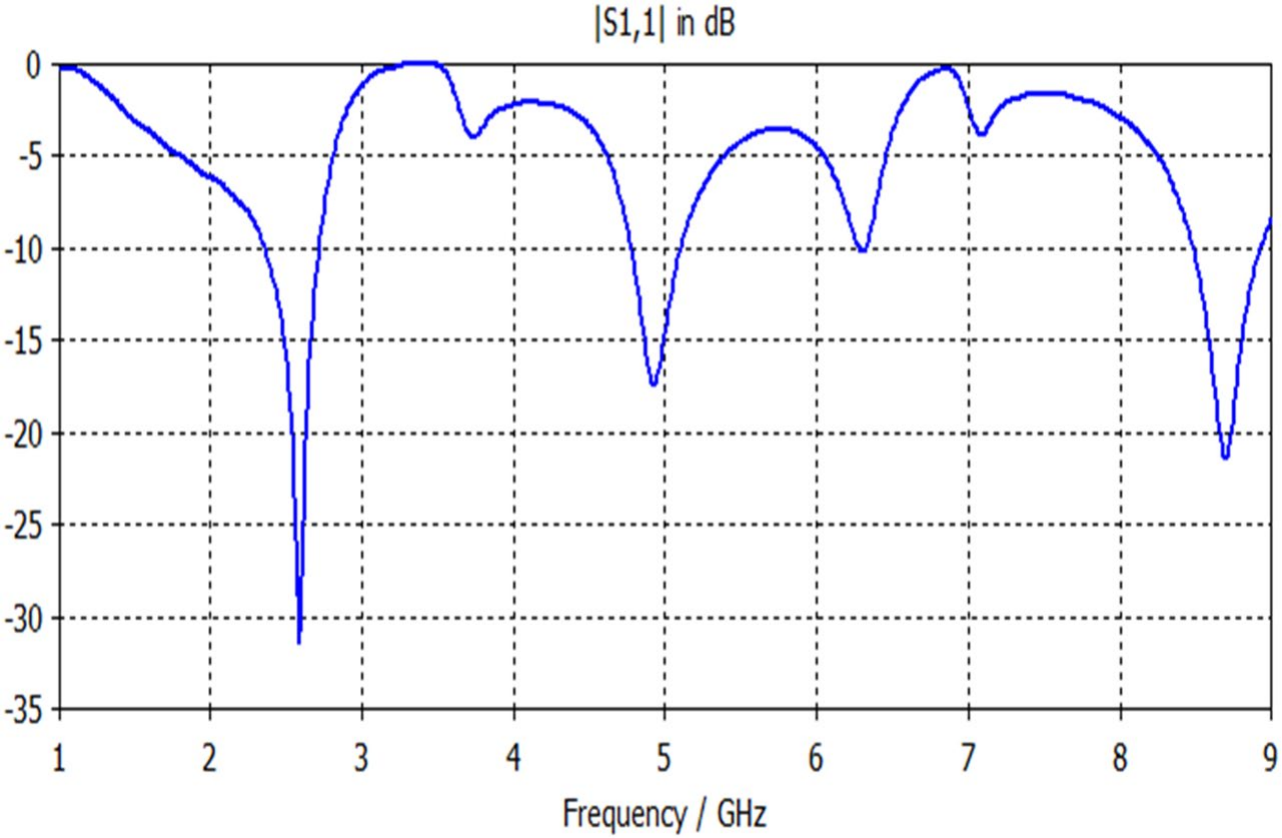
S11 response of Antenna2.

The input reflection response for Antenna2 is shown in Fig. 6. This antenna offers multi-band responses using a feedline with a length of 29 mm. The insertion of the octagon patch acts as an induction element which affects the appearance of the resonance. It is based on an uncomplicated Euclidean shape that behaves like the fractal or semi fractal antenna structures since it produces multiband responses that require specific formulas and fractal topologies with specified iterations^19^. This antenna is appropriate for WLAN and satellite applications. The magnitudes of frequency bands, their S11 values and bandwidths are shown in Table 2.

**Table 2:** Electrical properties of Antenna2 response.

**F1 (GHz)**	**2.55**
**F2 (GHz)**	5
**F3 (GHz)**	8.7
**S11 (F1) dB**	− 31.4
**S11 (F2) dB**	− 17.4
**S11 (F3) dB**	− 21.2
**BW1 (GHz)**	0.37
**BW2 (GHz)**	0.31
**BW3 (GHz)**	0.44

**Figure 7:**
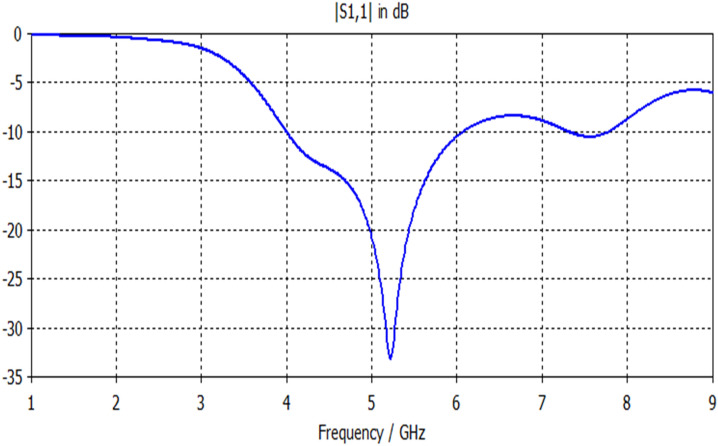
S11 response of Antenna3.

The S11 response of printed octagon based slot antenna (Antenna3) depicted in Fig. 3 can be observable from Fig. 7. The octagon based slot antenna offers a single band resonance with a wide bandwidth of 2 GHz suitable for WiMAX applications. Table 3 summarizes its performance.

**Table 3:** Electrical specification of Antenna3.

F1 (GHz)	S11 (F1) dB	BW (GHz)
5.2	− 33	2

**Figure 8:**
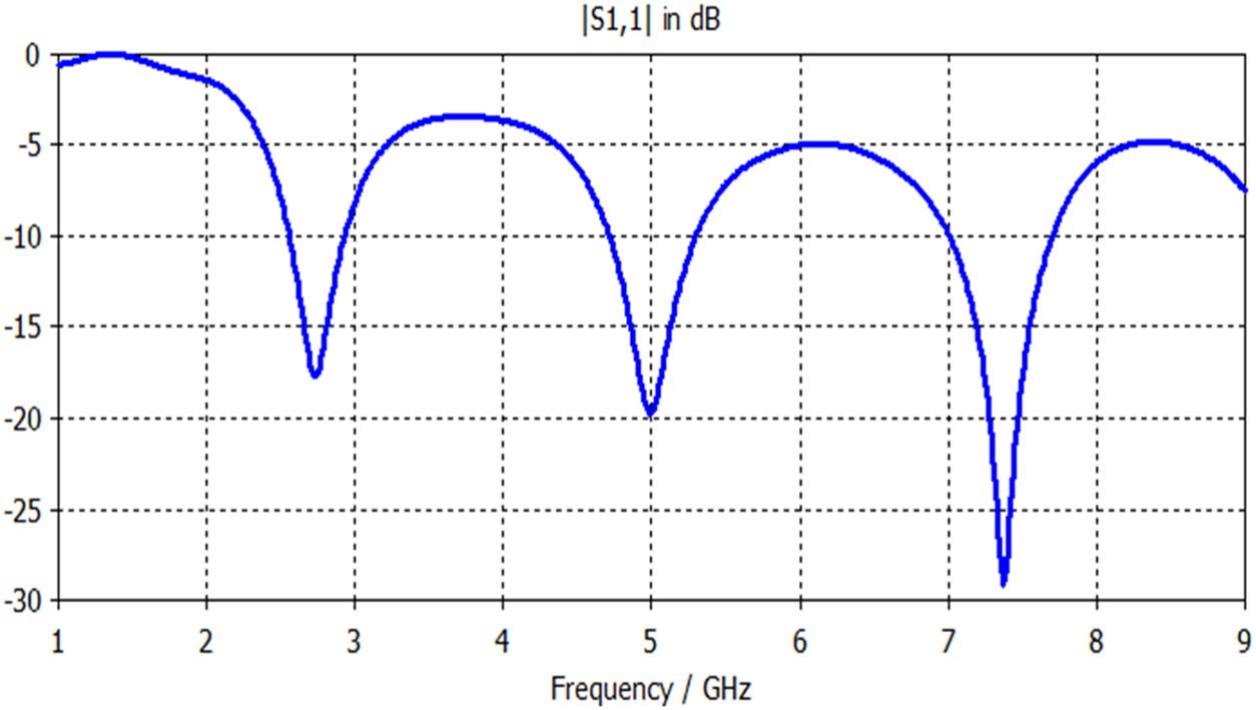
S11 response of Antenna4.

Lastly, the input reflection response of antenna based on an octagonal with circular patch (Antenna4) is depicted in Fig. 8. This antenna exhibits a multiband response, which is appropriate for WiMAX, WLAN, and satellite applications. As seen from this graph, the patch's adding acts as a perturbation element; this affects the appearance of multiple band resonances with lower bandwidths and input reflection values compared with Antenna3. Table 4 explains the details of Antenna4 response concerning resonant frequencies, input reflection values at these band frequencies as well as its bandwidths.

**Table 4:** Electrical properties of Antenna 4.

F1 (GHz)	2.74
**F2 (GHz)**	5
**F3 (GHz)**	7.4
**S11 (F1) dB**	− 17.6
**S11 (F2) dB**	− 19.7
**S11 (F3) dB**	− 29
**BW1 (GHz)**	0.37
**BW2 (GHz)**	0.6
**BW3 (GHz)**	0.68

**Figure 9:**
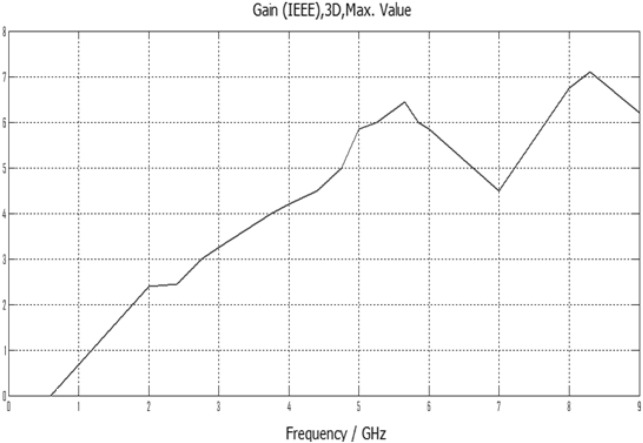
Gain result for Antenna1.

**Figure 10:**
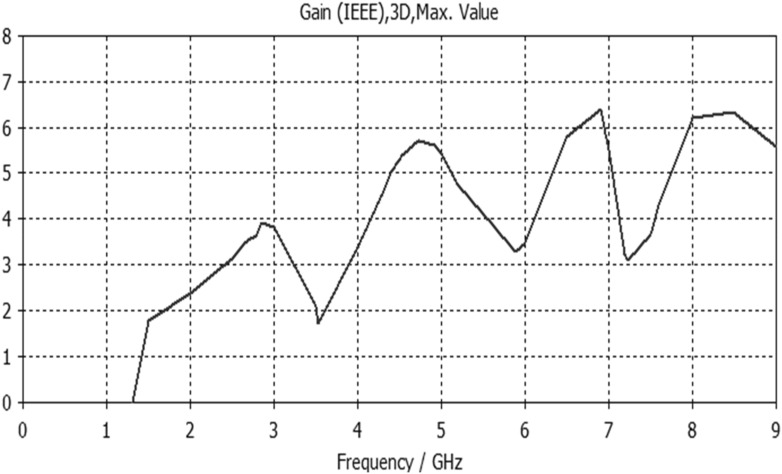
Gain result for Antenna2.

**Figure 11:**
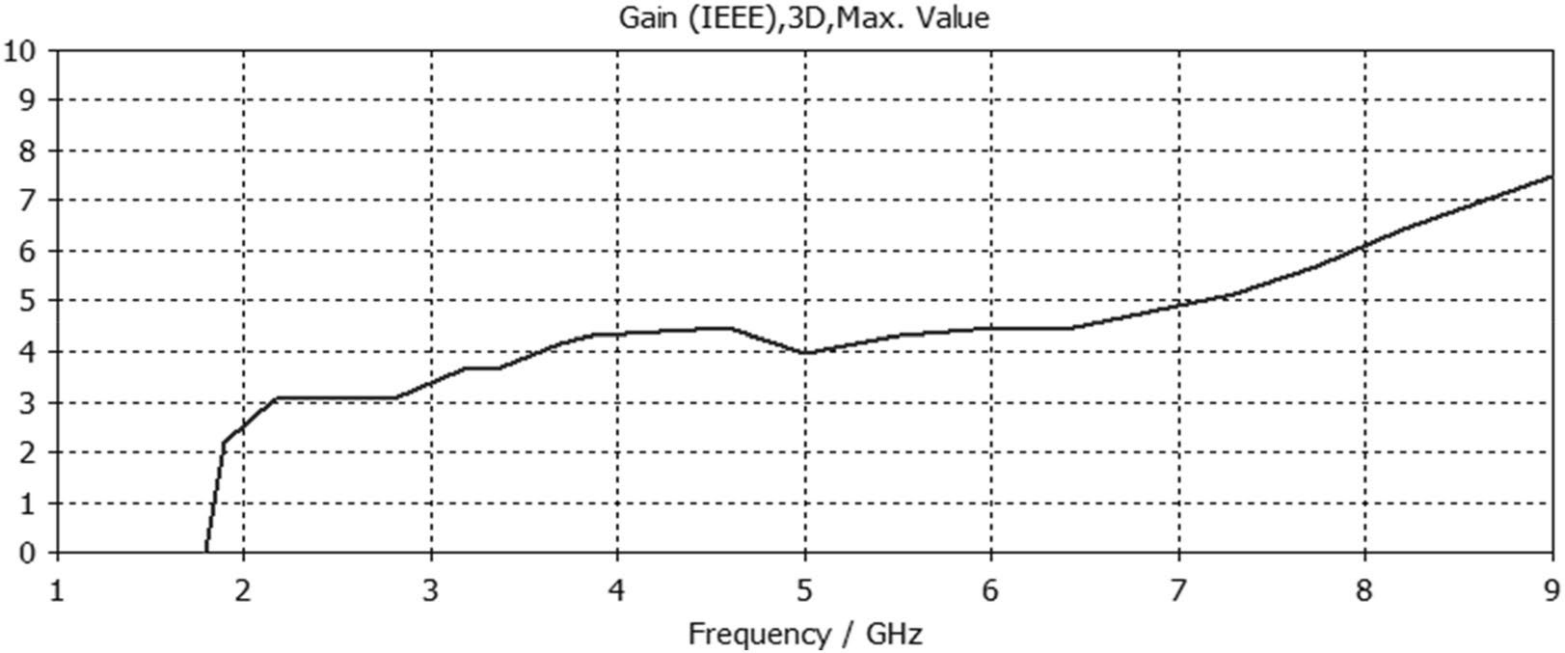
Gain result for Antenna3.

**Figure 12:**
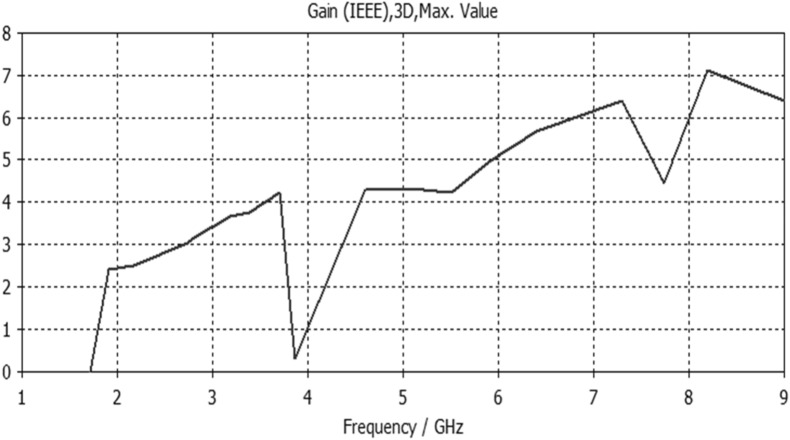
Gain result for Antenna4.

The IEEE gain magnitudes in units of dBi in the first elementary band have been evaluated in Figs. 9, 10, 11, 12 for Antennas1, 2, 3 and 4 correspondingly. These gain values are tolerable for typical wireless communication applications in use within these design frequencies. As frequency increases, the gain value is mostly increased primarily within (0–7) dBi.

**Figure 13:**
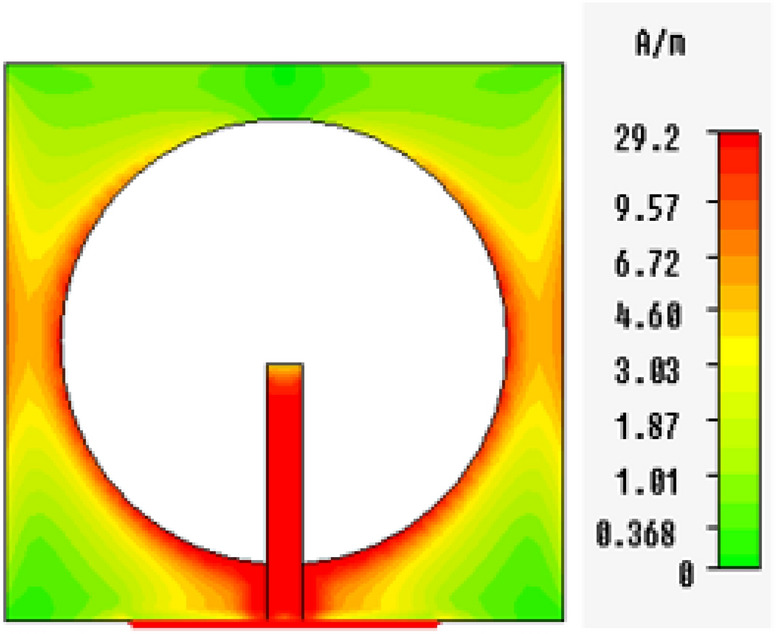
Magnetic intensity distributions for Antenna1 at the fundamental resonance of 2.7 GHz.

**Figure 14:**
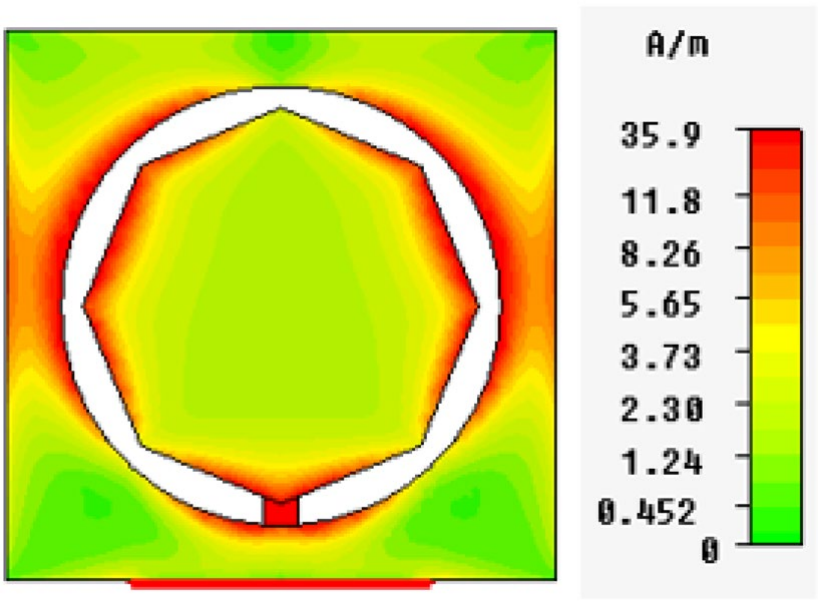
Magnetic intensity distributions for Antenna2 at the fundamental resonance of 2.55 GHz.

**Figure 15:**
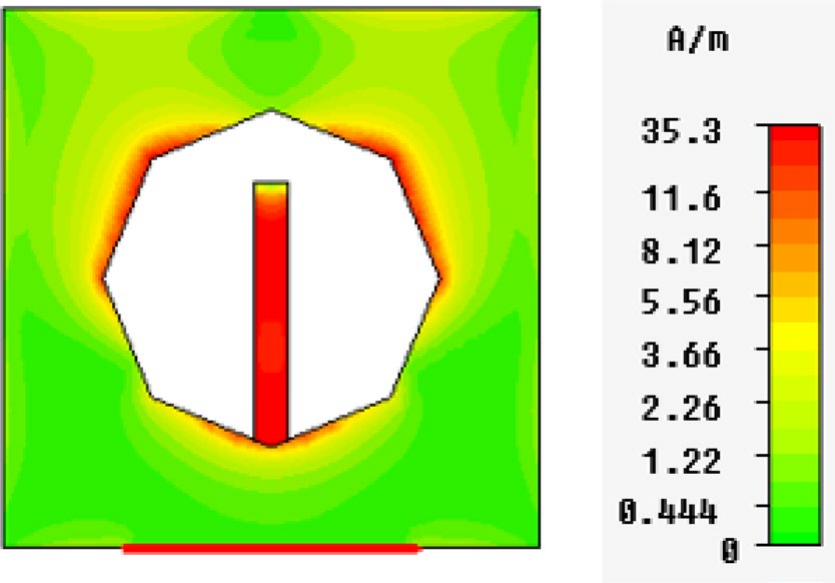
Magnetic intensity distributions for Antenna3 at the fundamental resonance of 5.2 GHz.

**Figure 16:**
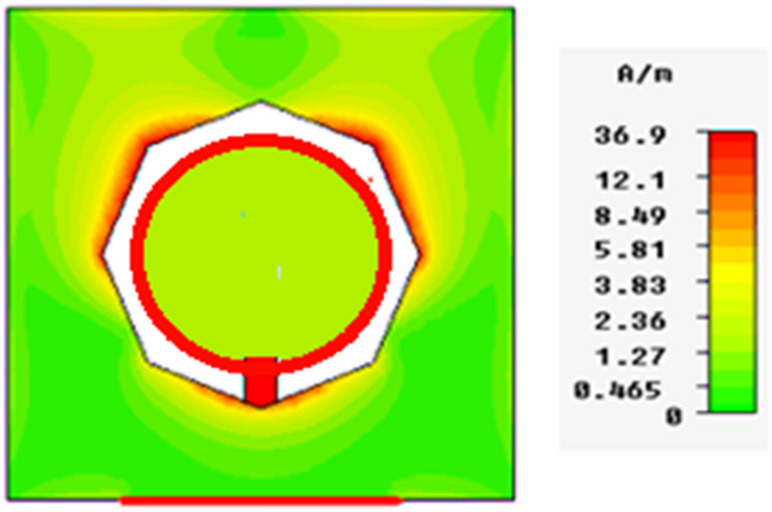
Magnetic intensity distributions for Antenna4 at the fundamental resonance of 2.74 GHz.

In Figs. 13, 14, 15, 16, magnetic intensity distributions are depicted based on the first (fundamental) resonance for each designed antenna to investigate the antennas' electromagnetic behaviors. The red color signposts the maximum magnetic intensity value, while the green color stands for the minimum. Namely, the effective areas are in regions with the highest magnetic intensity distributions. All distributions have been patterned symmetrically, and maximum magnetic intensity values are concentrated in the feed line, slot boundaries, and nearby regions. Based on these findings, feedline length and patch scaling aspects are adopted as useful factors in the parametric studies in Sect. 4 in this paper.

The proposed printed slot antennas in this study have been compared with other published papers in the literature regarding fractal antennas. Accordingly, Table 5 shows several performance parameters of several single-band, dual-band and multiband antennas manufactured by different fractal design approaches in reported studies. Among these parameters, a specific highlighting has focused on the antenna size, design principle, substrate material, resonances, and gain offered by the proposed antenna. Regardless of the number of bands and resonances, the projected printed slot antennas based on Euclidean geometries have competitive miniaturization and gain as compared with^20–27^. Also, the printed slot antenna designs presented in this paper have significantly simpler topologies and feeding methods than^20–27^.

**Table 5:** Comparison with other testified antennas in the literature.

Refs.	Size (mm^2^)		Design principle	Material	Resonances (GHz)	Gain (dBi)
^20^	50 × 50		Cantor fractal-based printed slot antenna	FR4	2.4/5.8	3.2/4.3
^21^	70 × 70		Dual reverse-arrow fractal	FR4	2.4	2.3
^22^	100 × 100		A dielectric resonator loaded Fractal slot loop antenna	FR4	1.42/2.61/3.65/4.93/6.15	2.2/2.2/3.4/4.4/4.0
^23^	55 × 60		Dragonfly fractal	Roger 5880	2.6/4.4/8.7	3.6/5.5/7.3
^24^	97*.*48 × 80		CPW Fed Fractal Antenna	FR4	2.39/3.77	4.56, 1.09
^25^	85 × 85		Microstrip fractal antenna	FR4	2.1/3.8/4.8	1.4/4.8/2.9
^26^	50.5 × 83.5		Perturbed Sierpinski monopole gasket	Arlon	1.1/3.4/5.8	1.74/5.95/4.22
^27^	78 × 38		Minkowski Fractal Antenna	FR4	1.54/1.88	…
Ant.1	50 × 50		Euclidean printed slot antenna	FR4	2.7/8.3	3.2/7.1
Ant.2	50 × 50		Euclidean printed slot antenna	FR4	2.55/5/8.7	3.1/5.5/6.4
Ant.3	50 × 50		Euclidean printed slot antenna	FR4	5.2	4.2
Ant.4	50 × 50		Euclidean printed slot antenna	FR4	2.74/5/7.4	3/4.3/7.4

## Parametric analyses

**Figure 17:**
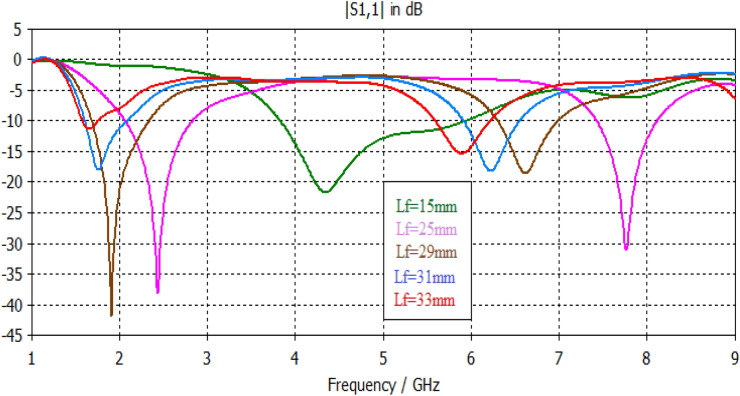
S11 responses of the antenna structure depicted in Fig. 1 with a feedline length as a parameter.

**Figure 18:**
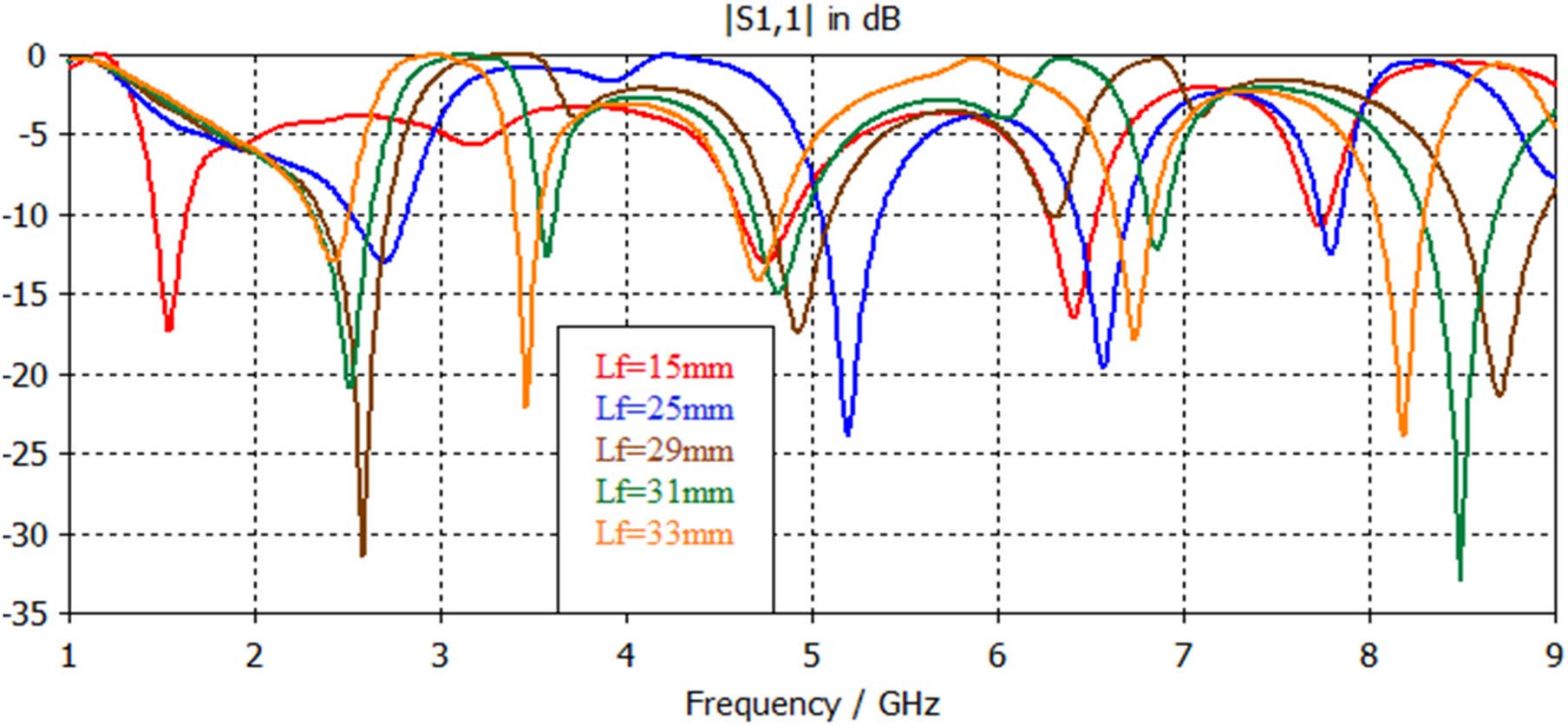
S11 responses of the antenna structure depicted in Fig. 2 with a feedline length as a parameter.

**Figure 19:**
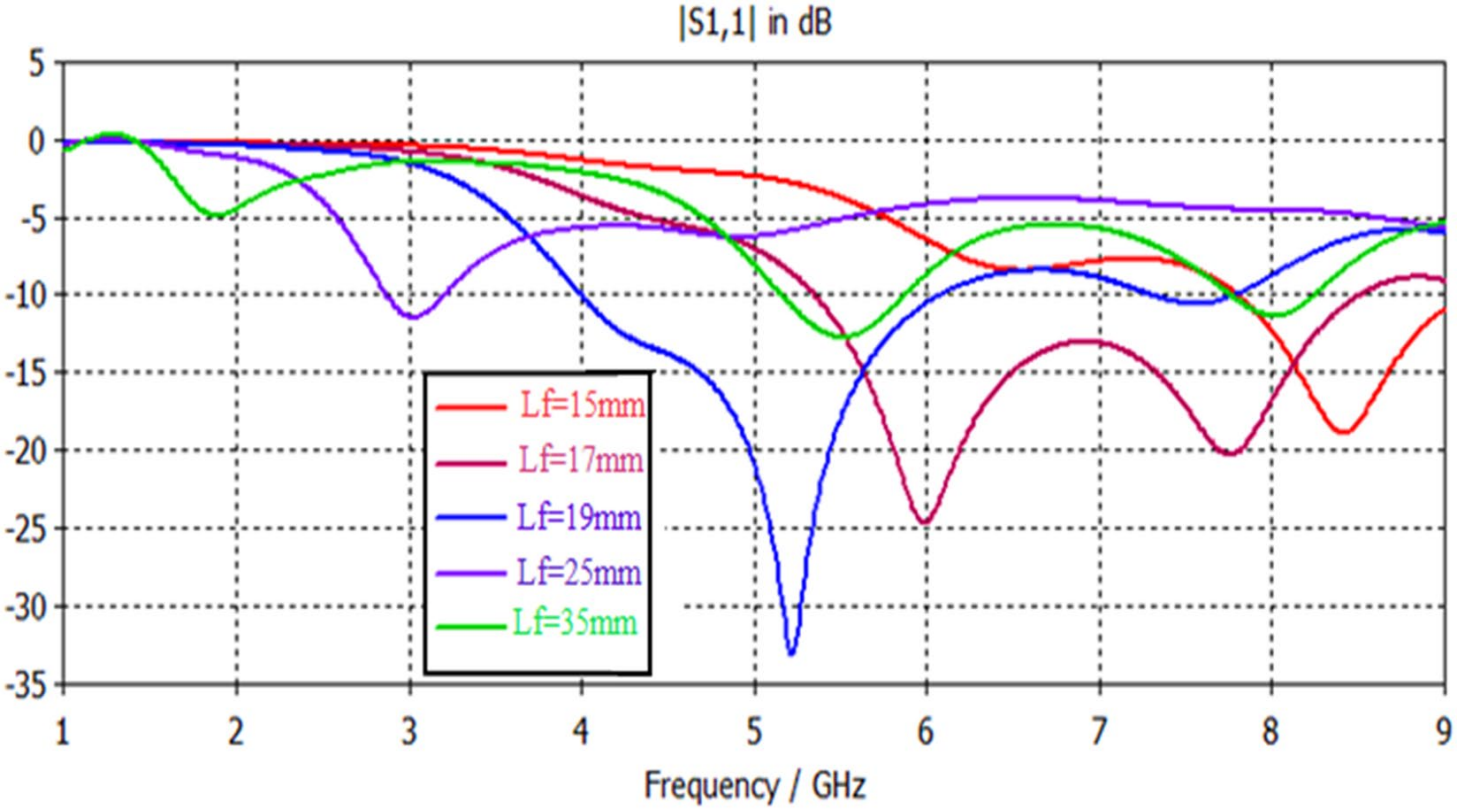
S11 responses of the antenna structure depicted in Fig. 3 with feedline length as a parameter.

**Figure 20:**
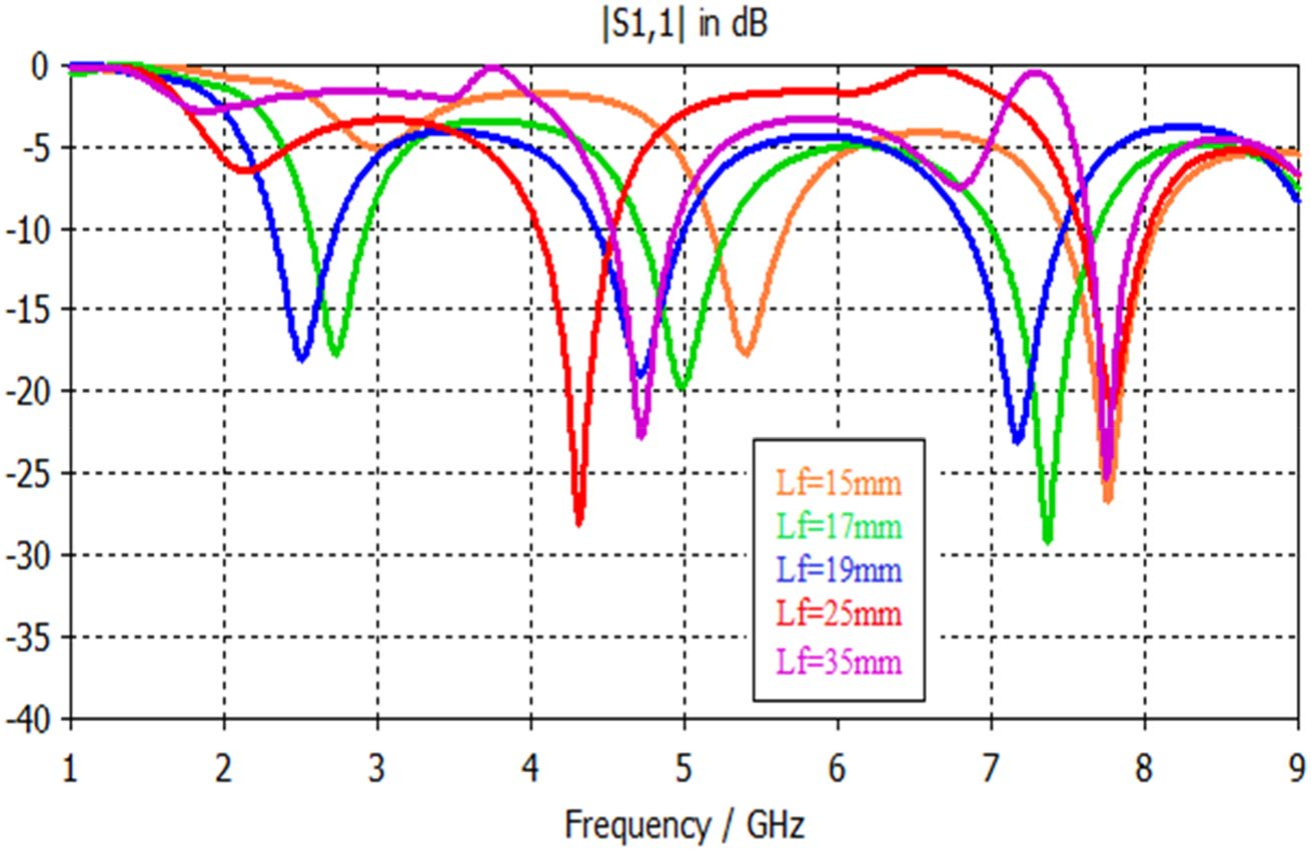
S11 responses of Antenna4 topology using a feedline length as a parameter.

**Figure 21:**
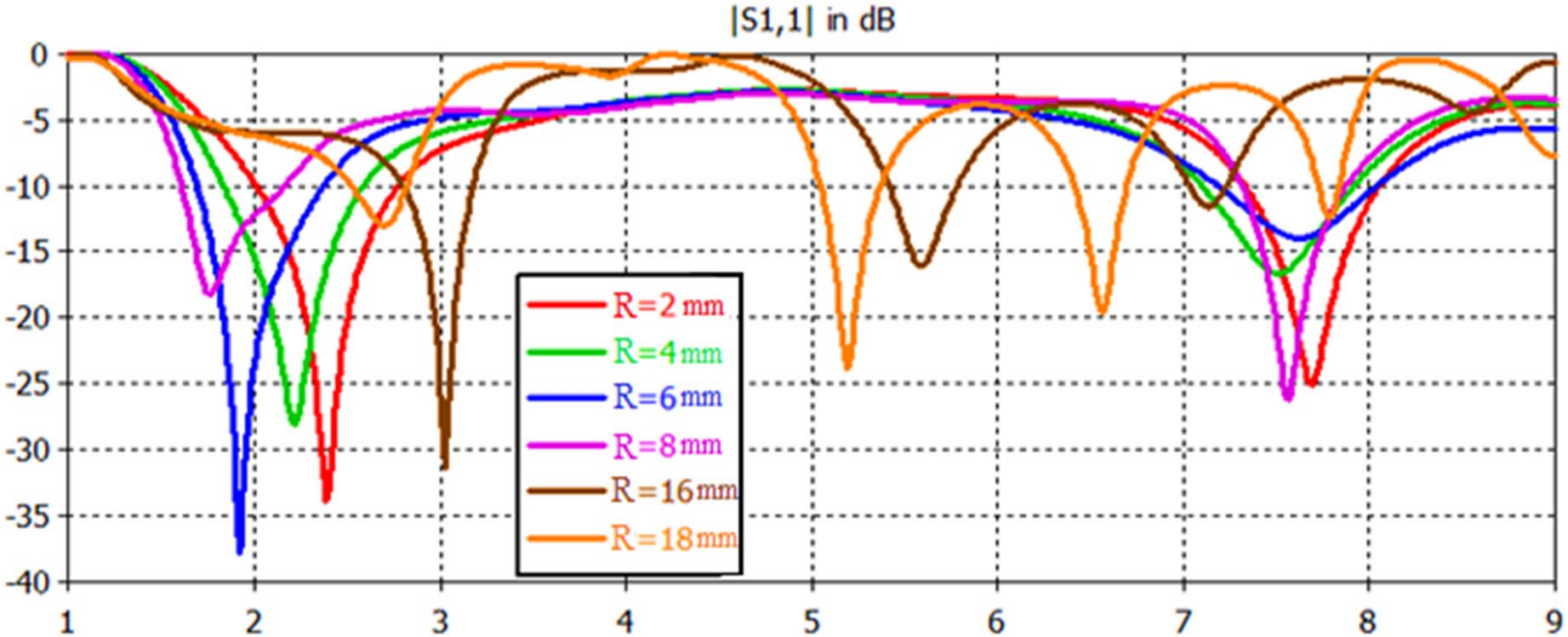
S11 responses for Antenna2 topology with respect to radius variations using a feedline length of 25 mm.

**Figure 22:**
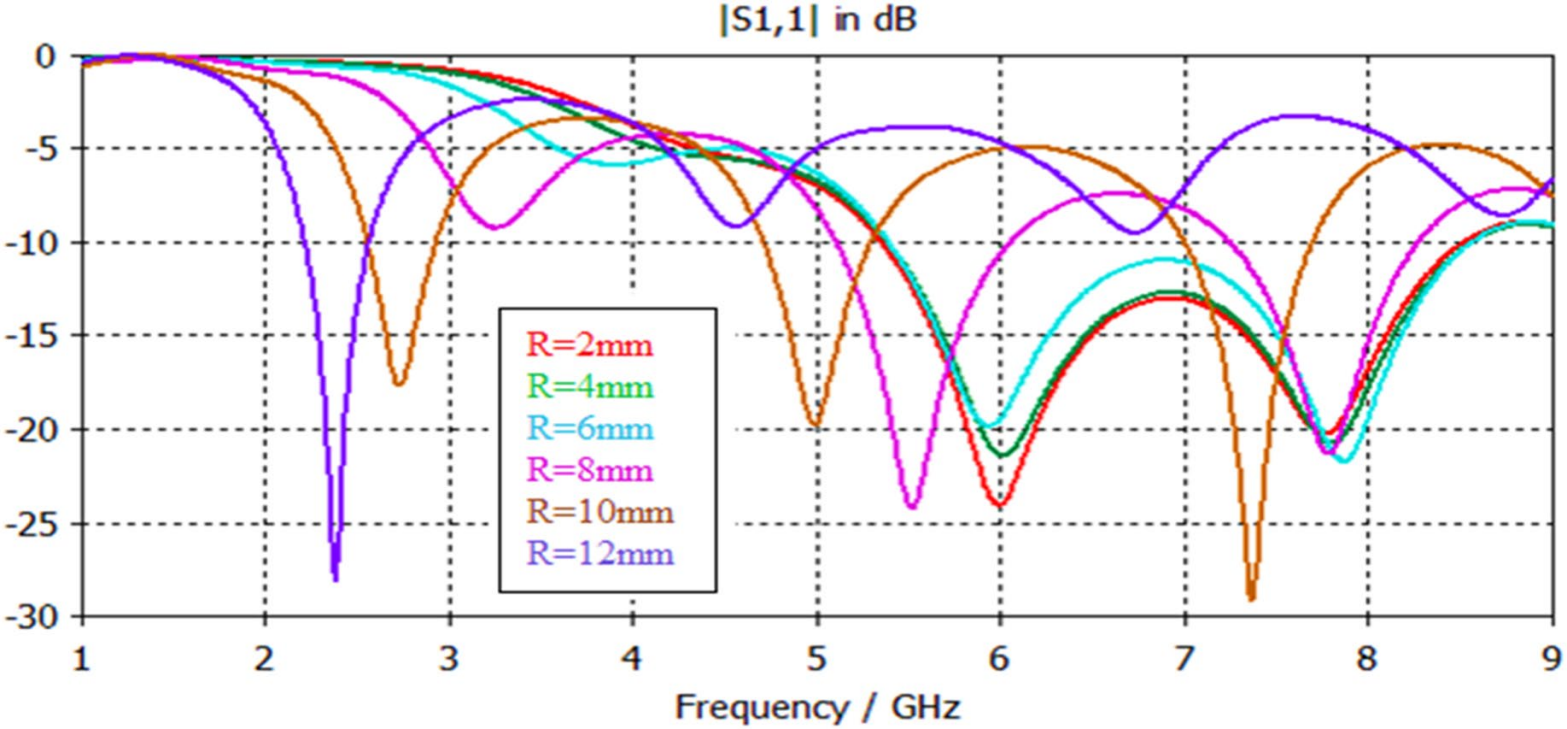
S11 responses based on Antenna4 patch radius variations using a feedline length of 17 mm.

Simulation results, shown in Figs. 17, 18, 19, 20, 21, 22, disclose that the modeled antenna can exhibit single, dual-band and multiband responses within the prescribed sweep frequency. This makes the antenna structure suitable for different wireless applications. In this case, simulation results show that the input reflections affected by two parameters; the feedline length and scaling factor of Euclidean patch structure. Parametrical investigations are achieved to discover the effects of these two parameters on the antenna performance with summarized electrical parameters.

### Effect of feed length

The feed line stands for a critical constituent that should be adjusted to work appropriately with the antenna. The microstrip feeder has been employed since it provides ease of fabrication and simplicity in modeling and impedance matching. Figures 17, 18, 19, 20 illustrate the projected antennas' input reflection results with respect to feedline length limitation. The feeder length significantly affects the antenna's frequency response in terms of resonances, number of bands, input reflection, and impedance bandwidth. For ranged feedline length from 15 to 35 mm, these antennas have potentially single, dual and multi-band responses with interesting features. Figure 17 demonstrates the input reflection response of the antenna depicted in Fig. 1 with feedline length as a parameter. Figure 18 illustrates the S11 responses of the antenna depicted in Fig. 2 with feedline length as a parameter. The parametrical responses of Fig. 17 have shown only single and dualband responses, while in Fig. 18, multiband responses are recognizably based on adopted feedline length. Tables 6, 7 clarify all electrical details of parametrical responses of Figs. 17, 18, respectively.

**Table 6:** Electrical properties of Antenna1 using a feedline length as a parameter.

Feed length (mm)	F1 (GHz)	F2 (GHz)	S11 (F1) dB	S11 (F2) dB	BW1 (GHz)	BW2 (GHz)
15	5	…	− 22	…	2.1	…
25	2.42	7.76	− 38.4	− 31.5	0.76	0.71
29	2	6.6	− 42.3	− 18.9	0.65	0.7
31	1.8	6.2	− 18.1	− 18.1	0.47	0.66
33	1.65	6	− 11.2	− 15.3	0.18	0.65

**Table 7:** Electrical properties of Antenna2 with respect to a feedline length as a parameter.

Feed length (mm)	15	25	29	31	33
**F1**	1.54	2.6	2.55	2.5	2.4
**F2**	4.7	5.2	5	3.5	3.4
**F3**	6.4	6.5	…	4.8	4.7
**F4**	7.72	7.7	8.7	6.8	6.7
**F5**	…	…	…	8.4	8.1
**S11 (F1) dB**	− 17.2	− 13.1	− 31.6	− 21.1	− 13
**S11 (F2) dB**	− 13	− 24.1	− 17.6	− 12.8	− 22
**S11 (F3) dB**	− 16.3	− 19.6	…	− 15	− 14
**S11 (F4) dB**	− 10.7	− 12.6	− 21.2	− 12.4	− 17.6
**S11 (F5) dB**	…	…	…	− 32.7	− 24
**BW1 (GHz)**	0.15	0.32	0.36	0.3	0.2
**BW2 (GHz)**	0.28	0.32	0.3	0.1	0.15
**BW3 (GHz)**	0.27	0.31	…	0.26	0.26
**BW4 (GHz)**	0.1	0.1	0.46	0.11	0.21
**BW5 (GHz)**	…	…	…	0.4	0.3

Figure 19 demonstrates the antenna's various input reflection responses in Fig. 3 with feedline length as a parameter. The parametrical responses of Fig. 19 have exposed only single and dualband responses. Table 8 explains their electrical specifications. On the other hand, Fig. 20 and Table 9 show the feasible multiband responses for Antenna4 topology.

**Table 8:** Electrical properties of Antenna3 with a feedline length as a parameter.

Feed length (mm)	F1 (GHz)	F2 (GHz)	S11 (F1) dB	S11 (F2) dB	BW1 (GHz)	BW2 (GHz)
15	8.4	…	− 19	…	1.2	…
17	7	…	− 24	…	3.13	…
19	5.11	7.6	− 33.2	− 10.8	2	0.38
25	3	…	− 11.5	…	0.31	…
35	5.5	8	− 12.8	− 11.7	0.7	0.44

**Table 9:** Electrical properties of Antenna4 topology using a feedline length as a parameter.

Feed length (mm)	F1 (GHz)	F2 (GHz)	F3 (GHz)	S11 (F1) dB	S11 (F2) dB	S11 (F3) dB	BW1 (GHz)	BW2 (GHz)	BW3 (GHz)
15	5.4	7.7	…	− 17.7	− 26.8	…	0.46	0.62	…
17	2.7	5	7.4	− 18.2	− 20	− 29.3	0.38	0.6	0.64
19	2.5	4.7	7.2	− 18.3	− 19	− 23.2	0.41	0.58	0.65
25	4.3	7.8	…	− 28.3	− 21	…	0.47	0.48	…
35	4.7	7.7	…	− 22.7	− 26	…	0.4	0.33	…

### Effect of antenna patch radius variation

Figure 21 and Table 10 depict the S11 results of Antenna2 topology presented in Fig. 2 with respect to antenna patch radius variation. For feedline lengths of 25 mm, the antenna has feasibly dual and multiband responses with interesting features. Figure 22 and Table 11 depict the proposed antenna's input reflection results shown in Fig. 4 with respect to antenna patch radius variation. For feedline lengths of 17 mm, the antenna has possibly single, dual and multiband responses with interesting features and wider impedance bandwidths than in Fig. 21.

**Table 10:** Electrical parameter details for Antenna2 with respect to patch radius variations using a feedline length of 25 mm.

Patch radius (mm)	2	4	6	8	16	18
**F1 (GHz)**	2.4	2.2	2	1.8	3	2.6
**F2 (GHz)**	7.6	7.5	7.6	7.6	5.6	5.2
**F3 (GHz)**	…	…	…	…	7.1	6.5
**F4 (GHz)**	…	…	…	…	…	7.7
**S11 (F1) dB**	− 34	− 28	− 38	− 18.5	− 31.1	− 13.3
**S11 (F2) dB**	− 25	− 16.4	− 14.2	− 26.5	− 16.4	− 24.3
**S11 (F3) dB**	…	…	…	…	− 12	− 19.5
**S11 (F4) dB**	…	…	…	…	…	− 12.8
**BW1 (GHz)**	0.75	0.75	0.7	0.58	0.37	0.32
**BW2 (GHz)**	0.73	0.76	0.77	0.5	0.36	0.3
**BW3 (GHz)**	…	…	…	…	0.21	0.36
**BW4 (GHz)**	…	…	…	…	…	0.14

**Table 11:** Electrical details of Antenna4 topology with respect to patch radius variations using a feedline length of 17 mm.

Patch radius (mm)	2	4	6	8	10	12
**F1 (GHz)**	7	7	7	5.5	2.7	2.3
**F2 (GHz)**	…	…	…	7.8	5	…
**F3 (GHz)**	….	……	……	……	7.3	…
**S11 (F1) dB**	− 23.6	− 21.3	− 21.3	− 24.3	− 17.5	− 28
**S11 (F2) dB**	…	…	…	− 21.1	− 20	…
**S11 (F3) dB**	…	…	…	…	− 29.3	…
**BW1 (GHz)**	3.13	3.13	3.13	0.92	0.33	0.35
**BW2 (GHz)**	…	…		0.98	0.62	…
**BW3 (GHz)**	…	…		…	0.65	…

## Measurement

**Figure 23:**
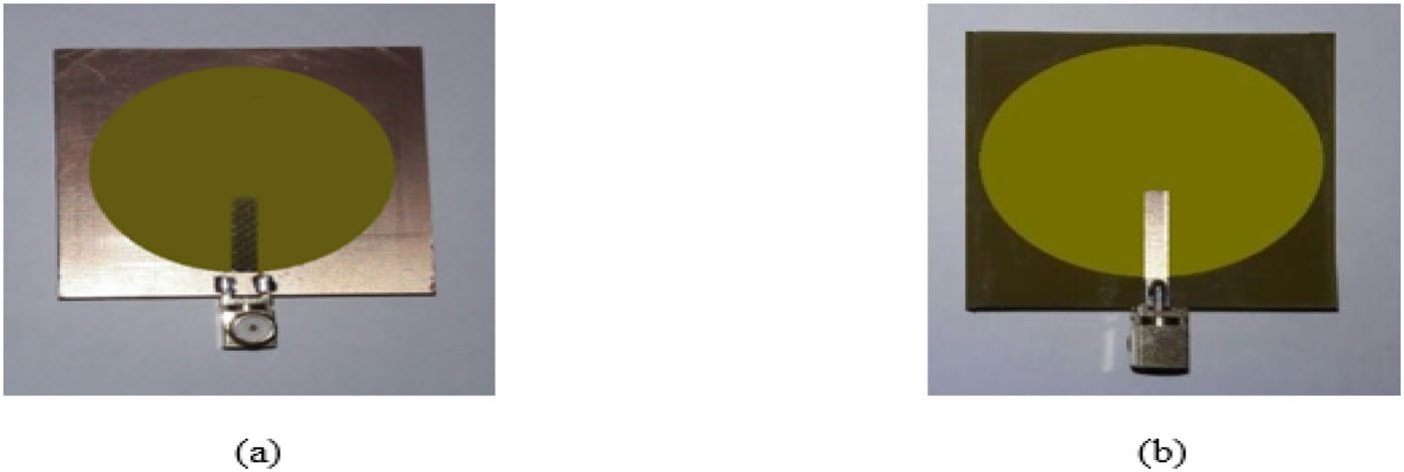
Manufactured prototype of Antenna1 (**a**) ground plane, (**b**) microstrip feed plane.

**Figure 24:**
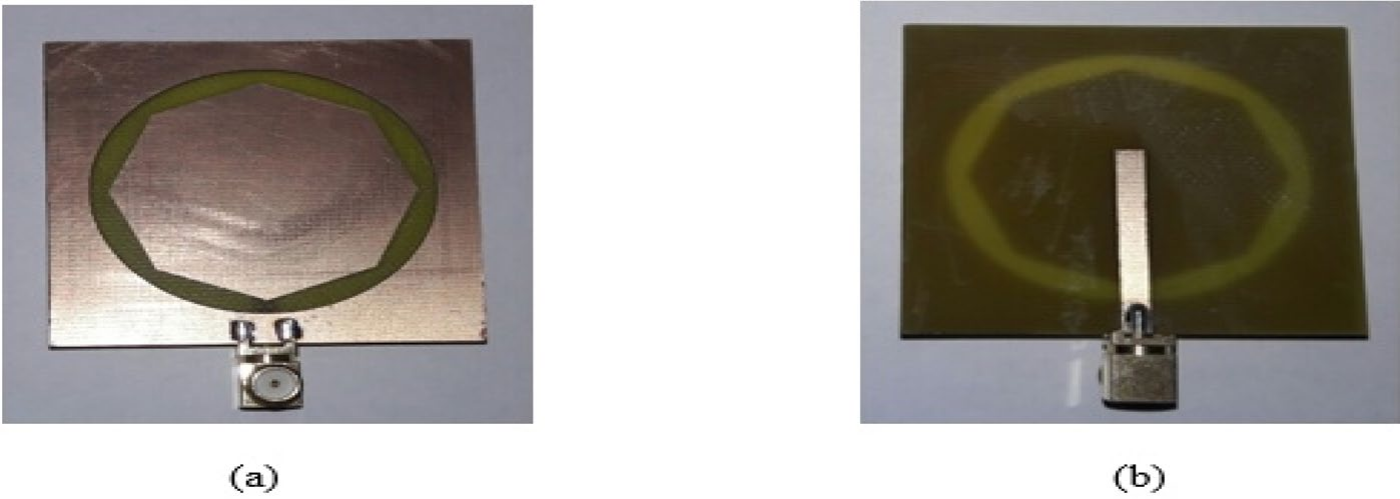
Manufactured prototype of Antenna2 (**a**) ground plane, (**b**) microstrip feed plane.

**Figure 25:**
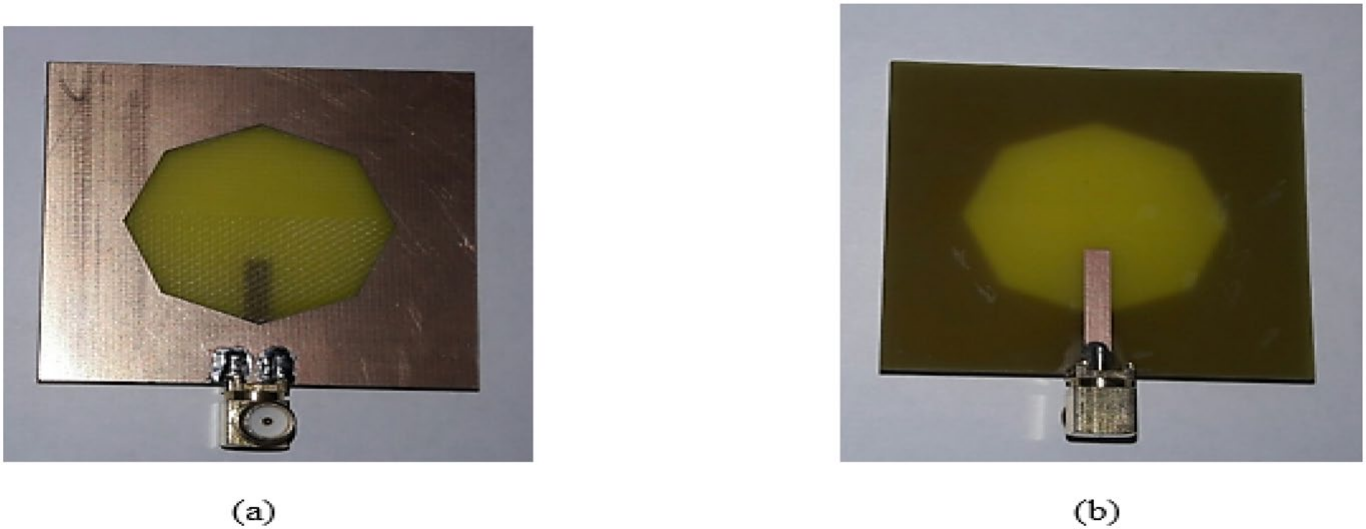
Manufactured prototype of Antenna3 (**a**) ground plane, (**b**) microstrip feed plane.

**Figure 26:**
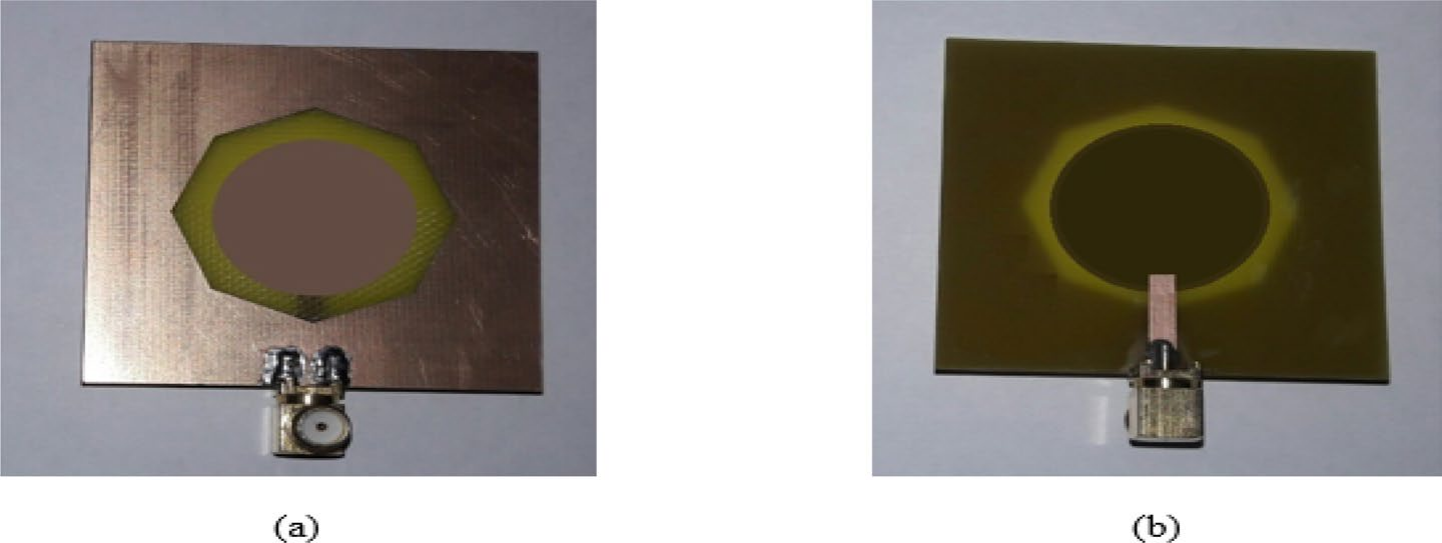
Manufactured prototype of Antenna4 (**a**) ground plane, (**b**) microstrip feed plane.

**Figure 27:**
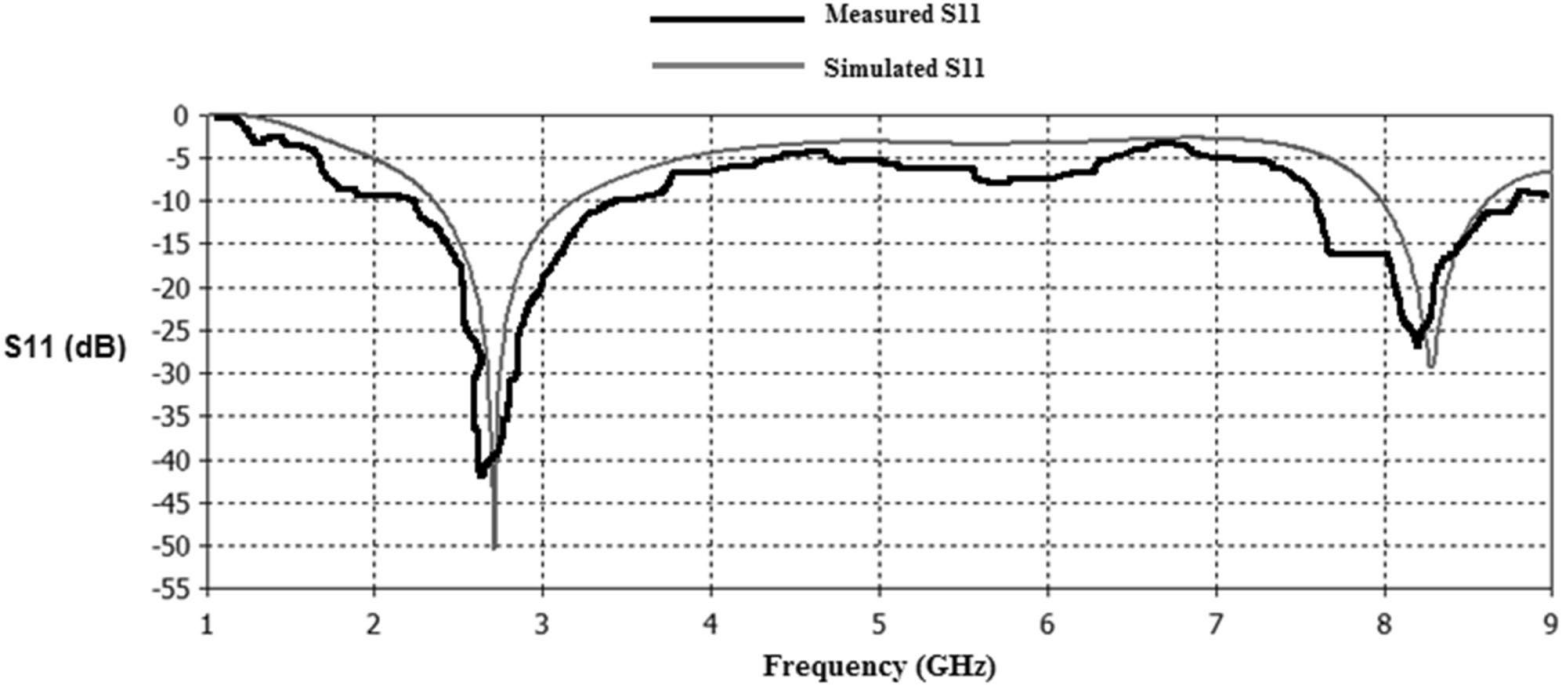
Simulated and measured S11 responses of Antenna1.

**Figure 28:**
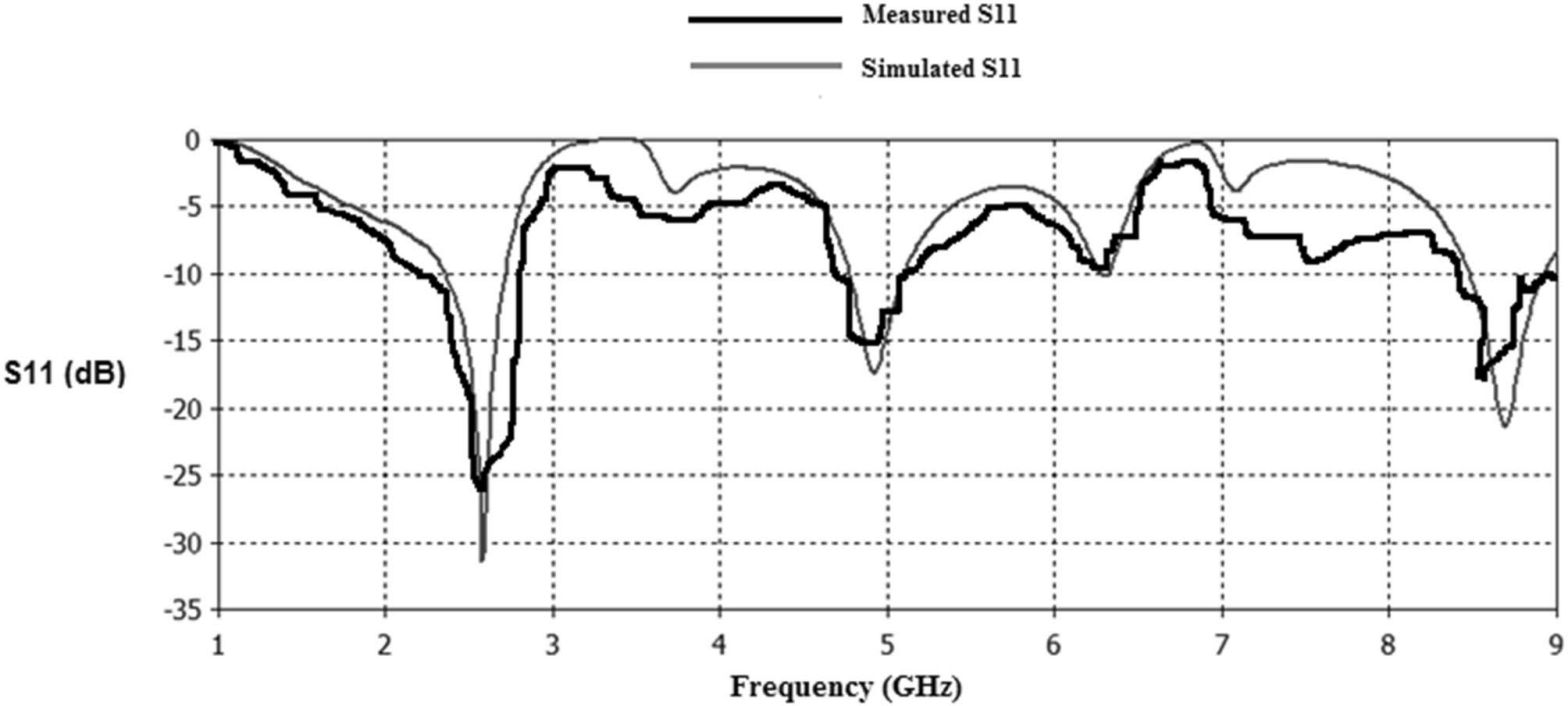
Simulated and measured S11 responses of Antenna2.

**Figure 29:**
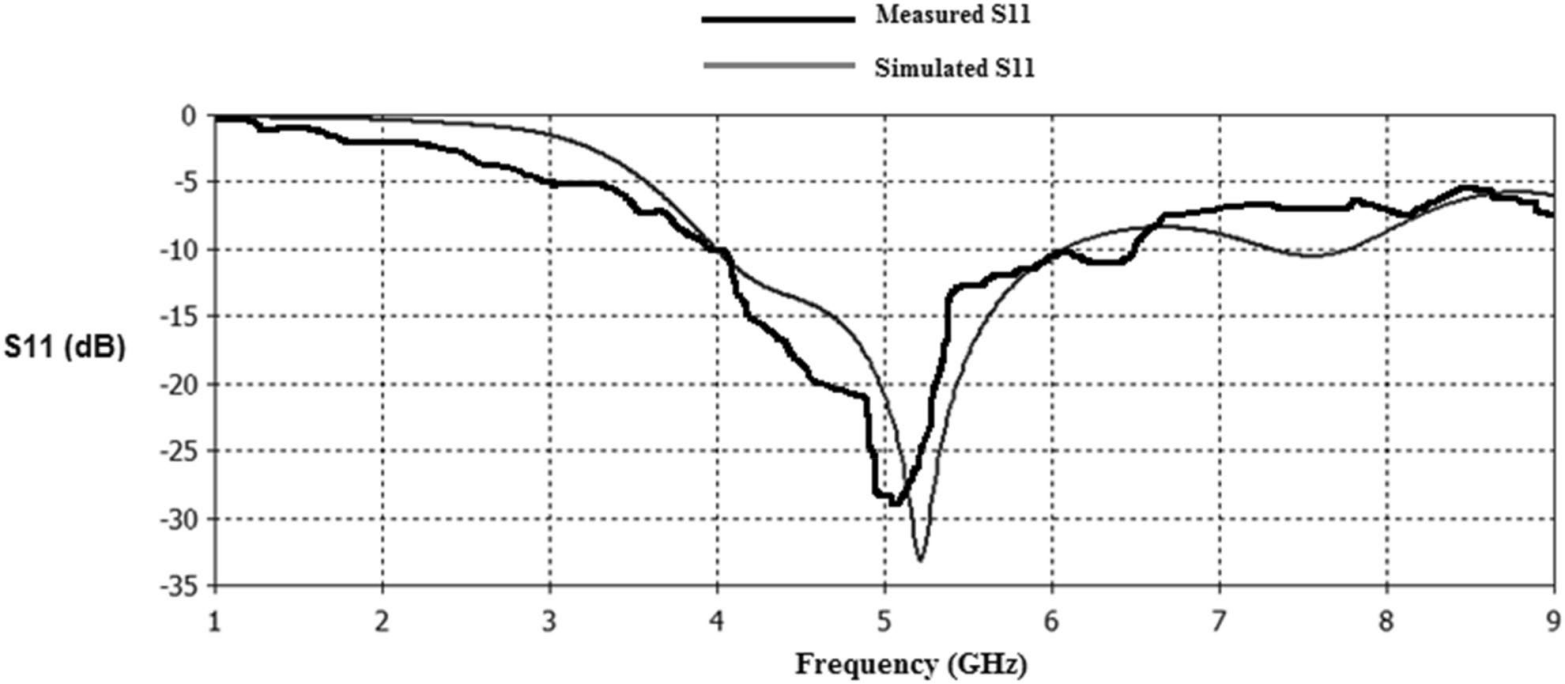
Simulated and measured S11 responses of Antenna3.

**Figure 30:**
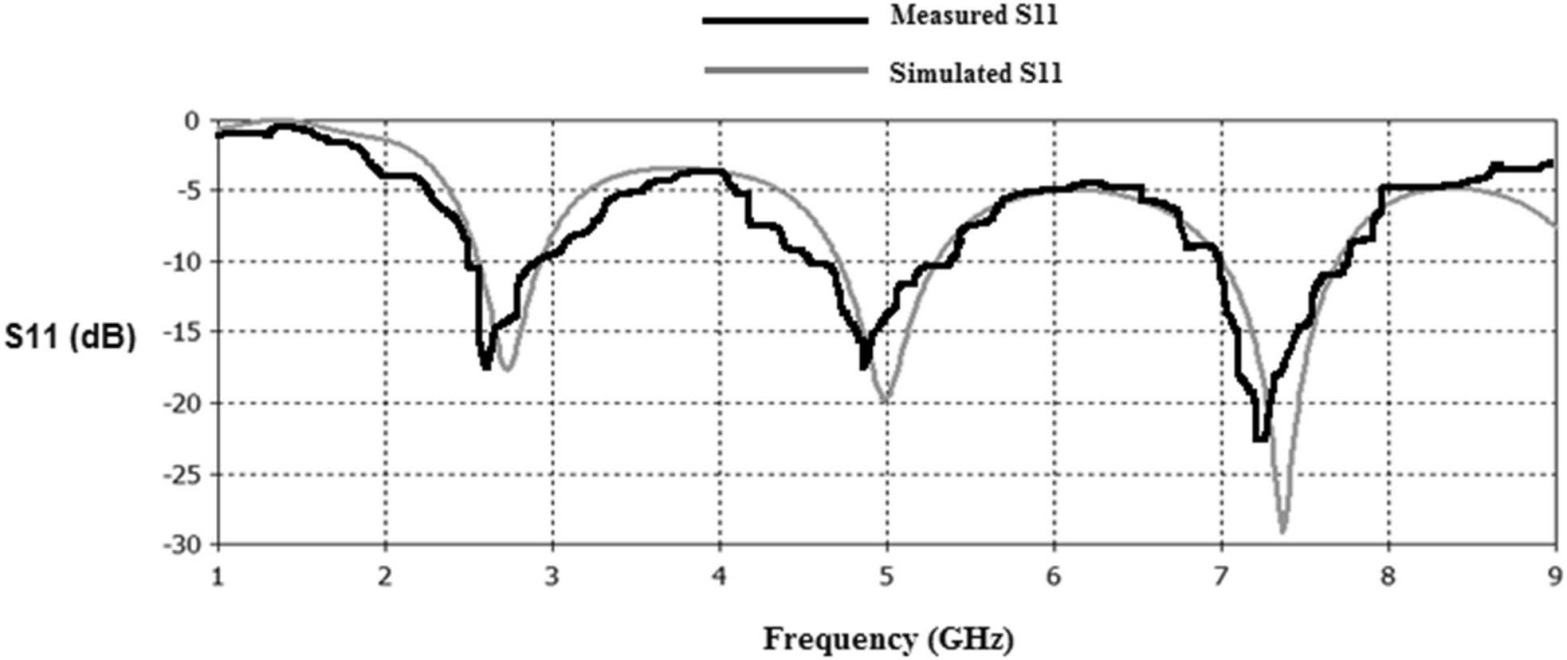
Simulated and measured S11 responses of Antenna4.

Figures 23, 24, 25, 26 illustrate the slotted ground structure and microstrip feed views of the fabricated prototypes of the designed Antennas1, 2, 3, and 4 employing LPKF machine. The measurements of S11 results have been done by Anritsu Vector Network Analyzer. The measured and simulated results of S11 responses are in acceptable agreement as shown in Figs. 27, 28, 29, 30. There are tolerable differences and ripples in the measurements as compared with the simulations due to manufacturing conditions, soldering and measurement environment. These differences are also related to a commercially cost-effective FR4 substrate used in this study for prototyping the printed slot antenna models. The substrate loss tangent becomes more in effect at the greater frequencies and results in more losses.

**Figure 31:**
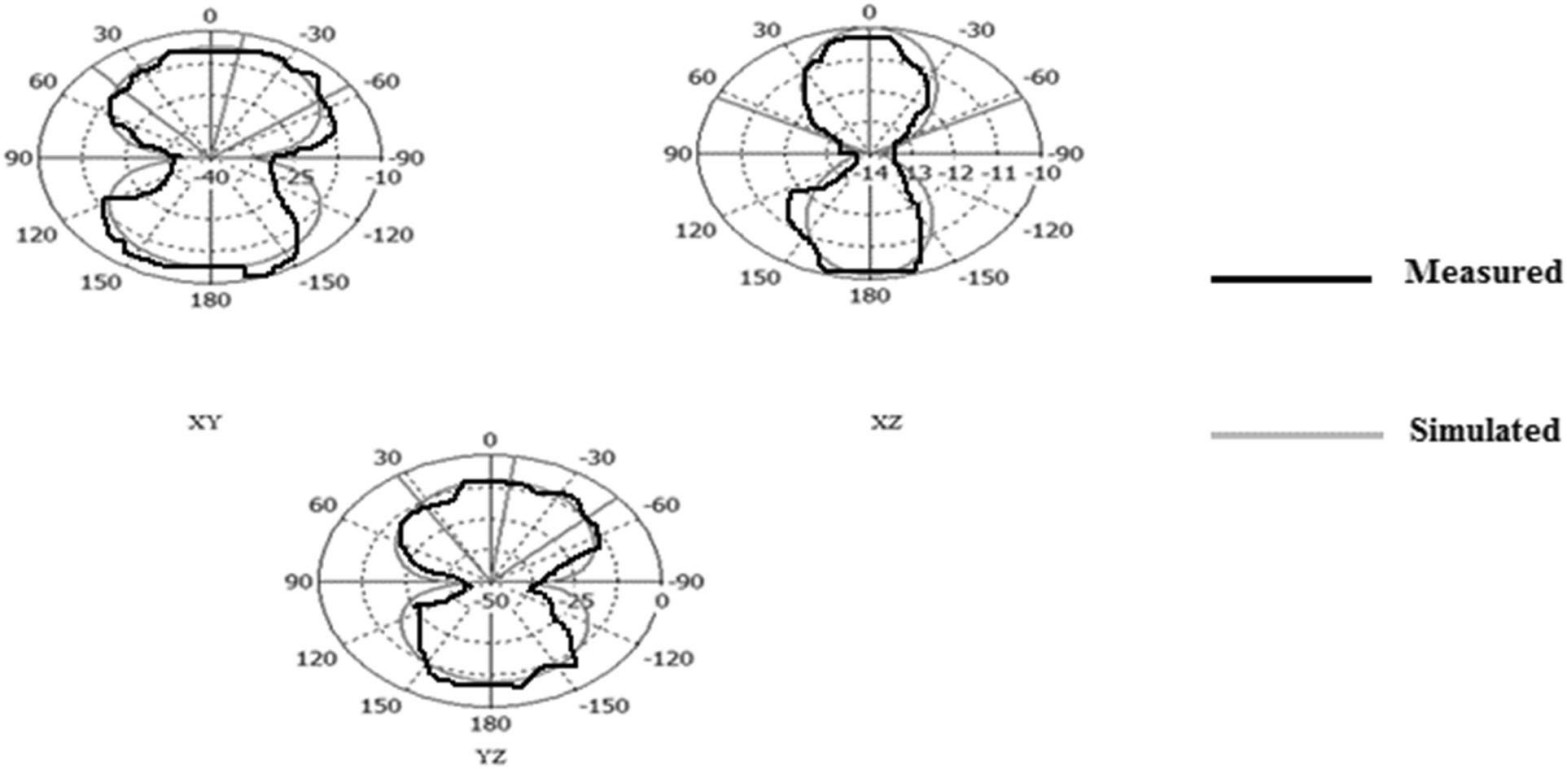
Simulated and measured radiation patterns of Antenna1 at the fundamental frequency of 2.7 GHz.

**Figure 32:**
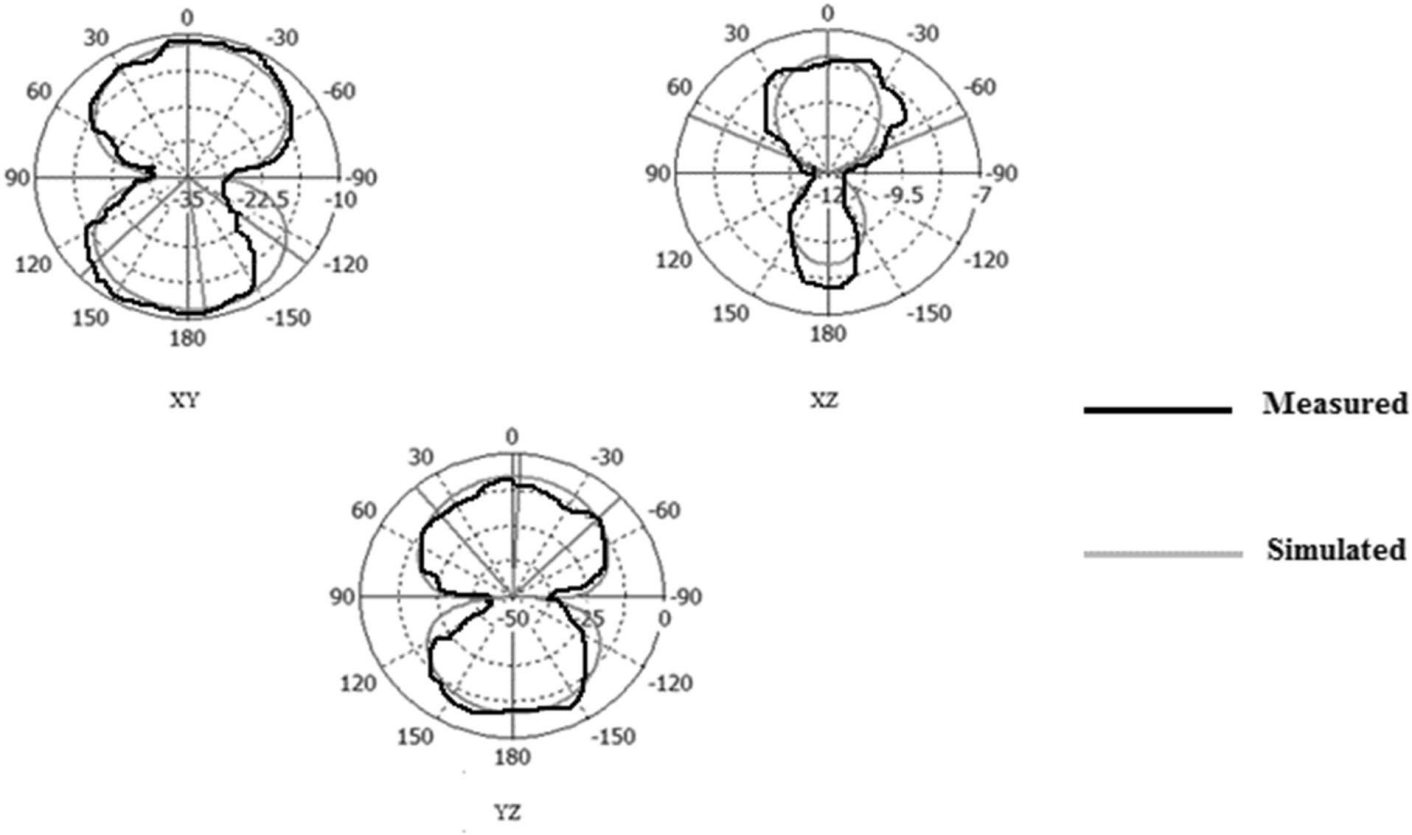
Simulated and measured radiation patterns of Antenna2 at the fundamental frequency of 2.55 GHz.

**Figure 33:**
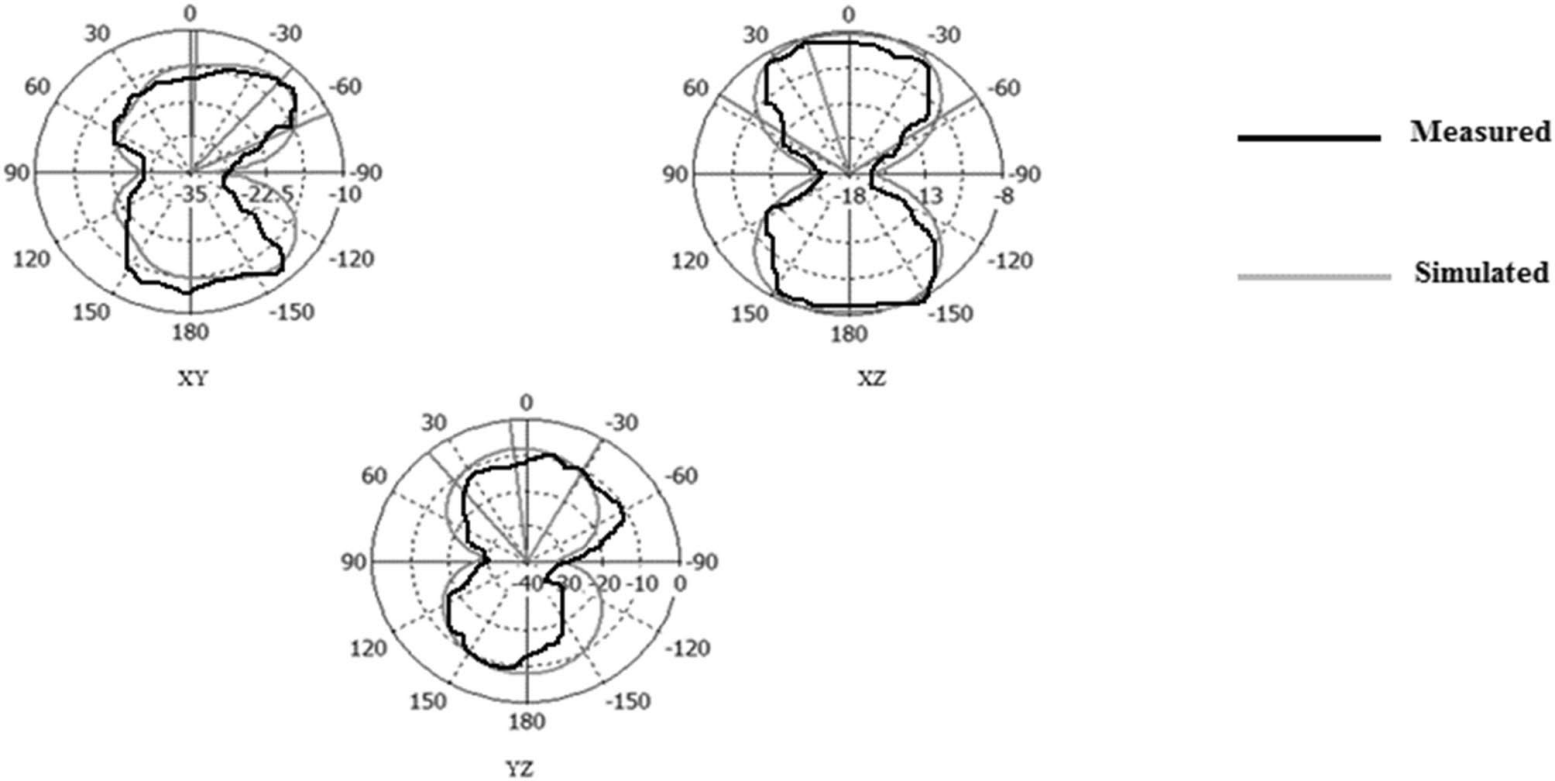
Simulated and measured radiation patterns of Antenna3 at the fundamental frequency of 5.2 GHz.

**Figure 34:**
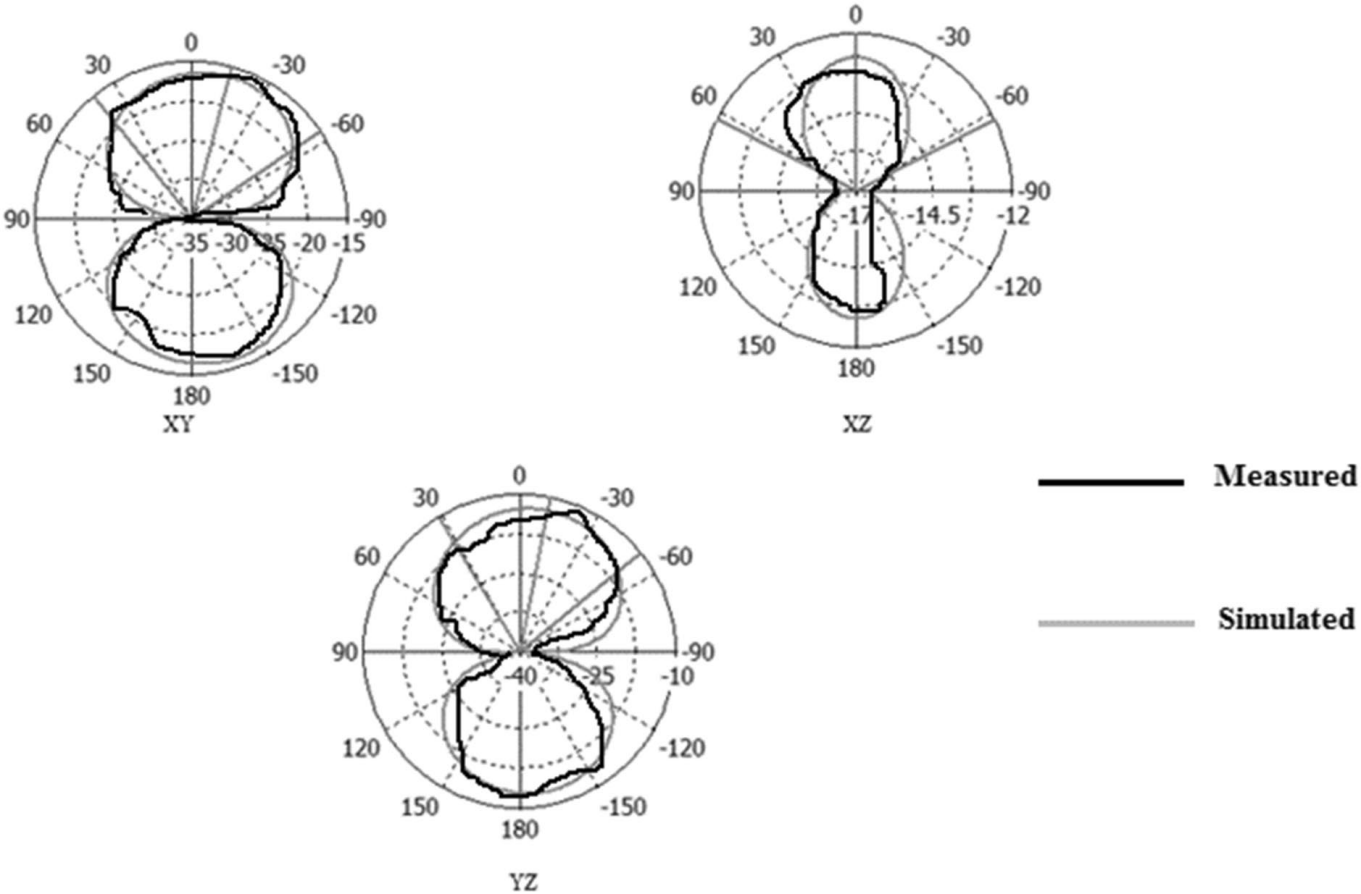
Simulated and measured radiation patterns of Antenna4 at the fundamental frequency of 2.74 GHz.

Figures 31, 32, 33, 34 describe the simulated and measured far-field radiation patterns for the total electric field in the x–y, x–z, and y–z planes for Antennas1, 2, 3, and 4, respectively, at their fundamental operating frequency. Both simulated and measured radiation patterns are in good agreement. For all radiation patterns, the x–y plane (θ = 90^o^, φ is variable), the x–z plane (φ = 0^o^, θ is variable) and the y–z plane (φ = 90°, θ is variable), they are all of bidirectional radiations patterns with tolerable performances and applicable for wireless applications.

## Conclusion

In this paper, four printed slot antennas have been introduced based on simple topologies of Euclidean slots with and without Euclidean patches. One of these antennas is a circle slot based structure with and without an octagon patch. These antennas produce dual and single-band responses for each of them, respectively. On the other hand, printed slot antennas using octagon slot based structure with and without circular patch are also presented. These antennas can produce dual and multiband responses with adequate performances. Parametrical predictions based on feedline length and radius of adopted patches on antenna performance can be employed to discover the consequences of these two parameters on the antenna performance with applicable electrical specifications. Printed slot antennas with Euclidean patches have potential multiband responses more than ones without Euclidean patches. However, printed slot antennas without Euclidean patches have wider impedance bandwidths, especially for single-band responses than ones with Euclidean patches. The proposed topologies of printed slot antennas are simpler than reported fractal antennas with competitive electrical parameters. Accordingly, these designed printed slot antennas can be incorporated within many portable and fixed wireless systems.

## Recommendation

The current work comprises of design and fabrication of printed slot antennas based on Euclidean structures for the GHz region. Many antennas for the THz region are being designed with improved Q-factor, which octagonal or hexagonal micro antennas may not display. However, different Euclidean structures can be investigated as a feasible future work to fix this issue.
